# Heritable epigenetic diversity for conservation and utilization of epigenetic germplasm resources of clonal East African Highland banana (EAHB) accessions

**DOI:** 10.1007/s00122-020-03620-1

**Published:** 2020-07-27

**Authors:** M. Kitavi, R. Cashell, M. Ferguson, J. Lorenzen, M. Nyine, P. C. McKeown, C. Spillane

**Affiliations:** 1 Genetics and Biotechnology Lab, Plant and AgriBiosciences Research Centre (PABC), Ryan Institute, National University of Ireland Galway, University Road, Galway, H91 REW4 Ireland; 2 International Institute for Tropical Agriculture (IITA), P.O. Box 30709-00100, Nairobi, Kenya; 3 Crop R&D, Agricultural Development, Bill & Melinda Gates Foundation, PO Box 23350, Seattle, WA 98102 USA

## Abstract

**Key message:**

Genetically identical East African Highland banana (EAHB) clones are epigenetically diverse with heritable epialleles that can contribute to morphological diversity.

**Abstract:**

Heritable epigenetic variation can contribute to agronomic traits in crops and should be considered in germplasm conservation. Despite the genetic uniformity arising from a genetic bottleneck of one ancestral clone, followed by subsequent vegetative propagation, East African Highland bananas (EAHBs) display significant phenotypic diversity potentially arising from somatic mutations, heritable epialleles and/or genotype-by-environment interactions. Here, we use DNA methylation profiling across EAHB accessions representing most of the primary EAHB genepool to demonstrate that the genetically uniform EAHB genepool harbours significant epigenetic diversity. By analysing 724 polymorphic DNA methylation sites by methylation-sensitive AFLP across 90 EAHB cultivars, we could differentiate the EAHB varieties according to their regions (Kenya and Uganda). In contrast, there was minimal association of DNA methylation variation with the five morphological groups that are used to classify EAHBs. We further analysed DNA methylation patterns in parent–offspring cohort, which were maintained in offspring generated by sexual (seed) and asexual (vegetative) propagation, with higher levels of altered DNA methylation observed in vegetatively generated offspring. Our results indicate that the phenotypic diversity of near-isogenic EAHBs is mirrored by considerable DNA methylation variation, which is transmitted between generations by both vegetative reproduction and seed reproduction. Genetically uniform vegetatively propagated crops such as EAHBs harbour considerable heritable epigenetic variation, where heritable epialleles could arise in offspring and contribute to functional traits. This study provides a basis for developing strategies for conservation of epigenetic resources and for integration of epimarkers into crop breeding programmes.

**Electronic supplementary material:**

The online version of this article (10.1007/s00122-020-03620-1) contains supplementary material, which is available to authorized users.

## Introduction

Future crop improvement and agricultural productivity increases are reliant on improved harnessing of the genetic diversity within primary crop genepools, and also within the secondary and tertiary genepools of crop wild relatives (Cooper et al. 2001; Halewood et al. 2018). Plant breeding programmes rely on access to crop germplasm resources consisting of elite breeding materials, existing varieties and germplasm accessions from primary crop and wild relative genepools (Castañeda-Álvarez et al. 2016; Rebetzke et al. 2018). Depending on the reproductive and propagation biology of the crop, genetic resources conservation programmes aim to conserve as much phenotypic and genetic diversity as possible in ex situ conservation programmes (e.g. in seed genebanks, in vitro clonal collections or field genebanks). Maximizing both phenotypic and genetic diversities represented within genebanks is a key goal for genetic resources conservation, which is enabled by molecular genetic tools, phenotyping (e.g. descriptors) and approaches such as the establishment of core collections (Brozynska et al. 2016; Spillane and Gepts 2001; van Hintum et al. 2000).

Clonally propagated crops, such as East African Highland Bananas (EAHBs), pose particular challenges for crop genetic resources conservation. In such species, clonal propagation, typically via somatic propagules, is used to maintain field genebanks and/or in vitro clonal collections (De Langhe et al. 2016; Engelmann 2011). For many clonally propagated crops, seed-based conservation is difficult due to seed recalcitrance to cold storage, sterility issues due to polyploidy and the difficulty of maintaining stable genotypes using seeds generated by meiosis (Engelmann 2011). East African Highland Bananas (EAHBs), which are sexually sterile triploids, are a major staple crop of importance to food and livelihood security in the Great Lakes region of East Africa and are commonly divided into cooking and beer types based on phenotypic characteristics (Batte et al. 2019; Kitavi et al. 2016; Němečková et al. 2018). The EAHB germplasm that is used as a basis for global EAHB breeding and crop improvement efforts is stored in both in vitro collections (International Transit Center *Musa* Collection managed by Bioversity International and hosted by the Katholieke Universiteit Leuven) and in field genebanks in Uganda—Mbarara and Tanzania-Sedussu (Kitavi et al. 2016).

East Africa has traditionally been considered as a secondary centre for diversity for East African Highland bananas, based on the presence of multiple phenotypically distinct EAHB cultivars in the region. However, we have previously demonstrated that all EAHB cultivars are genetically uniform at the genomic DNA level, having arisen from a single ancestral clone introduced to Africa, that subsequently underwent population expansion by vegetative propagation by farmers (Kitavi et al. 2016). This raises the question of the underlying basis for the phenotypic diversity observed in vegetatively propagated EAHB cultivars, which has likely arisen due to selection by smallholder farmers for different attributes over multiple generations and geographies. As there is no evidence for seed-based propagation of EAHBs by farmers that would introduce meiotic recombination events, sources of useful and adaptive phenotypic variation for vegetatively propagated EAHBs could consist of rare genetic mutations, genotype-by-environment (G × E) effects, epigenotype-by-environment (epi-G × E) effects and/or epimutations (epialleles) arising in the somatic lineages that vegetatively propagated EAHB cultivars represent (Kaeppler et al. 2000; Spillane and McKeown 2014).

Transgenerational epigenetic inheritance can occur for both meiotic (e.g. seeds arising from sexual recombination) and mitotic (e.g. vegetative propagation of somatic propagules) transmissions of epialleles (Grossniklaus et al. 2013; McKeown and Spillane 2014; Paszkowski and Grossniklaus 2011; Spillane and McKeown 2014). In plants, examples of epialleles that have likely been inherited over multiple generations (Quadrana and Colot 2016) include the *Lcyc* epiallele in *Linaria vulgaris* (Cubas et al. 1999). Environmental stress signals encountered in earlier generations of apomictic lineages of dandelion or inbreeding rice lines have been shown to be associated with heritable changes in epigenetic regulation of small RNAs and DNA methylation (Morgado et al. 2017; Zheng et al. 2017). In addition, epigenetic recombinant inbred lines (epiRILs) of *Arabidopsis thaliana* have revealed a major contribution of epigenetic variation to phenotypic variation in epiRIL lines that are genetically identical (Cortijo et al. 2014; Kooke et al. 2015; Zhang et al. 2018).

While there are a multiplicity of epigenetic marks at the DNA and chromatin (histone) levels, DNA methylation is a key epigenetic mark which can be mitotically or meiotically transmitted between cells, and between generations (Wendte and Schmitz 2018). DNA methylation, where a methyl group is added to certain cytosine bases, has been the most intensely studied of epigenetic marks and occurs in plants in both symmetric (CG, CHG) and asymmetric (CHH) contexts (H = A, C, or T). Stable patterns of DNA methylation levels between successive generations can arise from the DNA methylation state of the parental cells being faithfully transmitted to the offspring either sexually via meiosis or asexually, as happens during vegetative propagation or apomeiosis (Eichten et al. 2011). Methylated cytosines in CG and CHG contexts allow for transmission between cells and generations of the methylation patterns based on the parental strand information (Lunerova-Bedrichova et al. 2008; Saze et al. 2003). For vegetatively propagated crops, such as EAHBs, DNA methylation-based epialleles arising in somatic cells and tissues can be transmitted to subsequent generations through vegetative propagation that maintains a somatic lineage based on mitotic cell divisions.

To determine whether the genetically uniform, yet phenotypically diverse, genepool of EAHB harbours epigenetic diversity at the DNA methylation level, we profiled cytosine methylation patterns at CCGG sites and characterized methylation patterns across 90 genetically near-identical triploid East African Highlands Banana (EAHB) cultivars, which are phenotypically classified into five morphological clusters. In addition, we investigated whether the heritability of DNA methylation patterns differs depending on whether the DNA methylation status transmission is via meiosis (i.e. sexual reproduction) versus mitosis (i.e. by vegetative propagation). Our findings provide a basis for greater consideration of epigenetic diversity in germplasm conservation, particularly of clonally propagated crops and for incorporation of epigenetic markers in breeding programmes of clonally propagated crops.

## Materials and methods

### Plant materials

Three sets of EAHB plant materials were used for this study. Set 1 consisted of ninety genetically near-identical but phenotypically distinct triploid EAHB cultivars (Kitavi et al. 2016) with six outgroup cultivars (Supplementary Table 1): three Plantains (AAB genome), two dessert bananas (AAA genome) and one unknown genome, where A indicates a set of chromosomes whose ancestry derives from *Musa acuminate*, while B is indicative of *Musa balbisiana* ancestry. Set 2 consisted of crossing materials used to assess the inheritance of DNA methylation patterns via meiosis: three triploid EAHB cultivars (Nakawere, Entukura and Enzirabrahima, used as female parental lines for meiotic crosses), a diploid wild accession (Calcutta4, used as the male parent for meiotic crosses), four tetraploid *F*_1_ hybrids (201K-1, 1438K-1, 660K-1 and 917K-1) and 52 secondary triploids (*F*_2_’s) generated from subsequent crossing of the *F*_1_s with different improved males (C.V rose, SH-3217, Kokopo, Long Tavoy, Malaccensis, SH-3362, SH-3142, 5610s-1 and 9128-3, Supplementary Table 2). Set 3 consisted of nine families of vegetative clones (mother plant and first cycle offspring) (Supplementary Fig. 1) which were used to investigate mitotic transmission of DNA methylation patterns (via vegetative propagation).

The plant materials used in this study were sampled from field genebanks at two different geographical locations, namely (1) 49 EAHB accessions sampled from the east and central African banana regional germplasm collection at Mbarara, Uganda (Hamilton et al. 2016), which stands at an altitude of 1410 m above sea level, longitude 30° 36′ 8.34″ E and latitude 0° 35′ 57.29″ S. The Mbarara field genebank contains a total of 182 EAHB accessions (planted in the year 2000), predominantly landraces from Uganda, most of which are varieties that have distinct phenotypes. The remaining 41 EAHB accessions were sampled from (2) the field genebank at the Kenya Agricultural Research and Livestock organization (KALRO) in Kisii which has an altitude of 1818 m above sea level, latitude: 0° 40′ 26 S and longitude 34° 46′ 20. This field genebank was established in 2003 and predominantly comprises landraces from Kenya, with some from Uganda. Only varieties with clearly distinct phenotypes were sampled. Taken together, the 90 EAHB accessions sampled from (1) and (2) represented circa 50% of the total global EAHB germplasm collection. In the field germplasm collection, each accession is represented by four plants, established from suckers, planted in a single row and with a spacing of 3 × 3 m within and between rows. 3–4-cm samples of the cigar leaves of the banana at the same developmental stage were collected from the tip of each field-grown plant (Supplementary Fig. 2), and DNA was extracted using a modified CTAB protocol of Dellaporta et al. (1983) and Mace et al. (2003).

### Methylation-sensitive amplification polymorphism (MSAP)

Cytosine methylation of DNA was analysed in EAHB cigar leaf samples using the Keyte et al. (2006) methylation-sensitive amplification polymorphism (MSAP) protocol, with some modifications. For each sample, 250 µl of genomic DNA was digested using either the *EcoR*I/*Hpa*II or *EcoR*I/*Msp*I restriction enzyme combinations. Ligated DNA fragments were used as templates for pre-amplification with E00 and H/M00 primers (Supplementary Table 3) and the products diluted tenfold for selective amplification with six pairs of three nucleotide selective Eco*R*1/*Hpa*II primer combinations (Supplementary Table 3). The selective primers were labelled at the 5′ end with either NED™ (yellow) or 6-FAM™ (blue) (https://www.appliedbiosystems.com), enabling post-PCR mixing of the products to make a cocktail containing 1.5 µl NED and 1.0 µl 6-FAM. GeneScan 500 LIZ internal size standard (0.012 µl), formamide (9 µl) and 1 µl of PCR product cocktail were run and fragments read with a ABI PRISM 3730 XL Genetic Analyser (https://www.appliedbiosystems.com).

### MSAP fragment scoring and data analysis

To ensure consistency between electrophoresis runs, a control sample was included in every run and its fragment profile compared with the other samples in the same run. The advanced peak detection algorithm was used, with light smoothing turned on and all other settings left at default. Holland et al. (2008) bin widths of 0.5 produced topologies with the best resolution; hence, this bin width was used for the final analyses. Only unambiguous, intense bands from 150 to 500 bp were scored (Caballero and Quesada 2010; Liu et al. 2012). The quality of each msAFLP fingerprint and bin was manually checked following Whitlock et al. (2008) and Markert et al. (2010) with slight modifications. We only considered fragments with relative florescence units greater than 100 to reduce background noise. Methylation-sensitive fragments were scored for each DNA sample from *EcoRI*/*Msp*I and *EcoRI*/*Hpa*II-digested DNA using Genemapper v4.1 (Applied Biosystems). Following the protocol of Fulneček and Kovařík (2014), four MH fragment pattern variants ‘+ +, − −, + −, − +', referring to the presence (+) or absence (−) of a fragment, were called. Interpretation of methylation-sensitive amplified polymorphism (MSAP) fragment profiles was based on Fulneček and Kovařík (2014).

### Status of DNA methylation among triploid EAHB cultivars

To assess the extent of cytosine methylation of the EAHB cultivars, MSAP scorings (from differential digestion) were used to infer global methylation level (i.e. the proportion of total cytosines that are methylated irrespective of their specific sequence context). Total DNA methylation levels were calculated with the *Msap*R package v1.1.9 (Pérez-Figueroa 2013). Individual fragments (loci) were classified based on their restriction pattern as methylation-susceptible loci (MSL) or non-methylated loci (NML): +/− as mCmCGG,  +/− as CmCGG, −/+ as indeterminate methylation of an internal CCGG site on an *Eco*RI and *Msp*I/*Hpa*II digested band (either mCCGG or CmCGG), or as non-methylated loci (+/+). Significance was determined using multi-sample nonparametric Kruskal–Wallis tests with one-way ANOVA and Dunn’s multiple comparison test. The diversity of the MSL and NML was assessed by calculating Shannon’s index of phenotypic diversity, *S*, derived from the Shannon–Weaver index (Shannon 1948) as $S = -\sum\nolimits_{i - 1}^{n} {p _{i} \log 2p_{i} }$, where *P*_*i*_ is the frequency of the band presence at the *i*th marker within the population) using *msap* v1.1.9. Overall epigenetic population differentiation, βST (based on MSL), was calculated in *msap* v1.1.8.

### Within and between morphological-group analysis

The level and diversity of methylation states in the morphological clusters were calculated in R software version 3.6.1. To investigate the patterns of epigenetic variation within and between the EAHB phenotypic groupings, PCoAs were constructed from the MSL and NML cultivar profiles using the *msap* v1.1.8 package in R (supported by Mantel’s test with 1000 permutations) and visually compared. Transformation of the multistate raw data matrix from the *EcoR*I/*Hpa*II and *EcoR*I/*Msp*I profiles into a binary data matrix allowed statistical analyses and computation of descriptive indices such as epigenetic diversity between cultivars. AMOVA analysis was applied to the pairwise distance matrix to partition the sources of the observed variation by component parts.

Multivariate analysis was used to explore epigenetic structure between morphological groups. Principal coordinate analyses (PCoA) were performed on inter-profile covariance matrices based on MSL and NML binary profiles (Liu et al. 2012). PCoA profiles were grouped into populations maximizing the between-group variance using between Eigen analyses (BPCA) (Lira-Medeiros et al. 2010; Liu et al. 2012; Parisod and Christin 2008). Statistical significance was assessed by the Romesburg randomization test (10^4^ permutations). Multivariate analyses were performed to obtain a PCoA using dudi.pco and s.class with the R package ade4 v1.7.13 (Thioulouse et al. 1997).

### Epigenetic population structure and relationships

The inherent epigenetic structure of EAHB population was investigated using the Pritchard et al. (2000) method that implements a model-based clustering method in the program STRUCTURE v2.3.4. Characterized by a set of allele frequencies at each locus, the EAHB cultivars were assigned to populations (*K*), identified as migrants or admixed using MSAP multilocus genotype data independent of prior population information (Pritchard et al. 2000). The number of possible Ks (1–10) was assessed in three replicates assuming the admixture model with correlated allele frequencies and 20 independent runs with 100,000 Markov Chain Monte Carlo (MCMC) iterations and 100,000 burn-in. Lambda was set at the program default of 1.0 for exploratory analyses without a priori information about individual origin. The most likely value of *K* was derived using the second-order rate of change *L*″(*K*) (Evanno et al. 2005; He et al. 2011; Lerceteau-Kohler et al. 2013) using the online tool Structure Harvester (https://taylor0.biology.ucla.edu/struct_harvest).

### Heritability of methylated loci in meiotic *F*_1_ hybrids versus mitotic progeny of East African Highland bananas

The level and diversity of methylation states in parents versus their offspring were calculated in R. Differentiation of the non-methylated and methylation-susceptible loci of the meiotic progeny versus the mitotic progeny was calculated based on the φst values of the loci. Methylation patterns present in both the parents and their corresponding offspring were regarded as inherited. Methylated-loci bands that were present in offspring and not in their parents were regarded as new methylation marks. Loss of methylation was scored where bands were present in the parents but missing in their respective offspring. Significant differences in methylation levels between parent(s) and subsequent generations were determined by one-way ANOVA with Tukey correction, as well as with the Kruskal–Wallis test and Dunn test of multiple comparison to determine differences between the methylation states across subdivisions within the data.

## Results

### Global DNA methylation variation across the EAHB genepool

The levels and diversity of methylation patterns in 90 East African Highland Banana (EAHB) cultivars and outgroups were determined using MSAP with the methylation-sensitive isoschizomeric restriction enzymes *Hpa*II and *Msp*I. The (+, +) pattern (a fragment visualized in both the Msp and Hpa profiles) was attributed to digestion by both enzymes at a non-methylated CCGG site. The (−, −) pattern indicated inhibition of digestion with both enzymes at a fully methylated mCmCGG site. The (+, −) pattern which represents a fragment of a defined size visualized in the *Msp*I but not in the *Hpa*II profile corresponds to digestion with *Msp*I but not *Hpa*II and indicates the presence of a CmCGG site (CG methylation). The (−, +) pattern observed in *Hpa*II but not in *Msp*I is difficult to interpret unambiguously in plant genomes (where methylation occurs in both the CCG and CG motifs) and is indicative of a CCGG site within the band: that is, it corresponds to either a CmCGG site or a mCCGG site (Fulneček and Kovařík 2014). MSAP data were combined into two datasets: Dataset 1 (from EAHB sample set 1) included 724 fragments and was used for the analysis of DNA methylation polymorphism and epigenetic structure of the EAHB population. Dataset 2 (from EAHB sample sets 2 and 3) included 1868 bands and was used to assess the trans-generational inheritance of DNA methylation patterns via meiosis (parental line, sexual crosses *F*_1_s crosses (tetraploids) and *F*_2_s) and mitosis (vegetative clones and first cycle offspring).

**Fig. 1:**
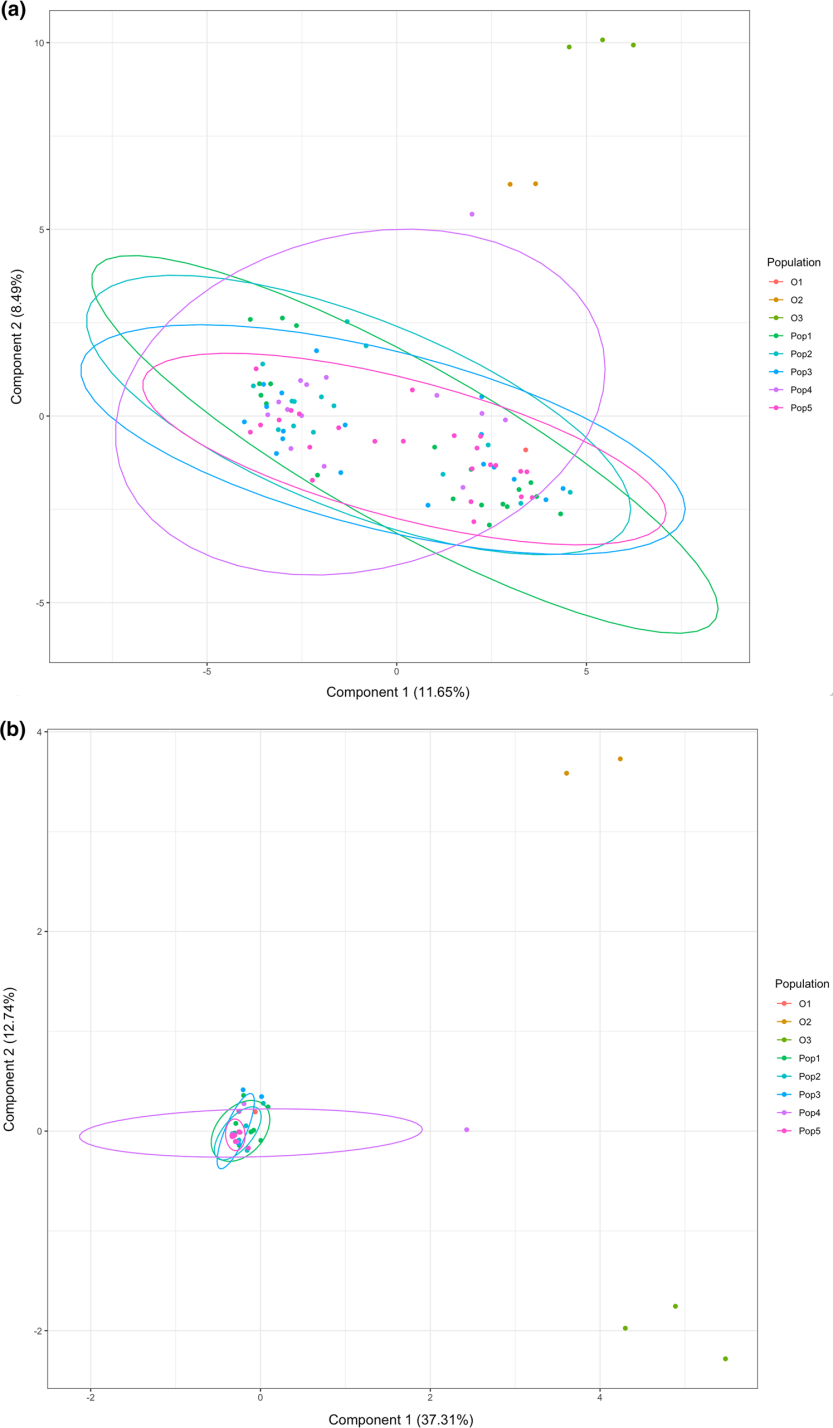
Principal coordinates analyses (PCoAs) illustrating variation between the 90 EAHB cultivars and six outgroup varieties (S/No. 1–96, Supplementary Table 1) based on **a** the methylated loci (MSL) and **b** non-methylated loci (NML)

**Fig. 2:**
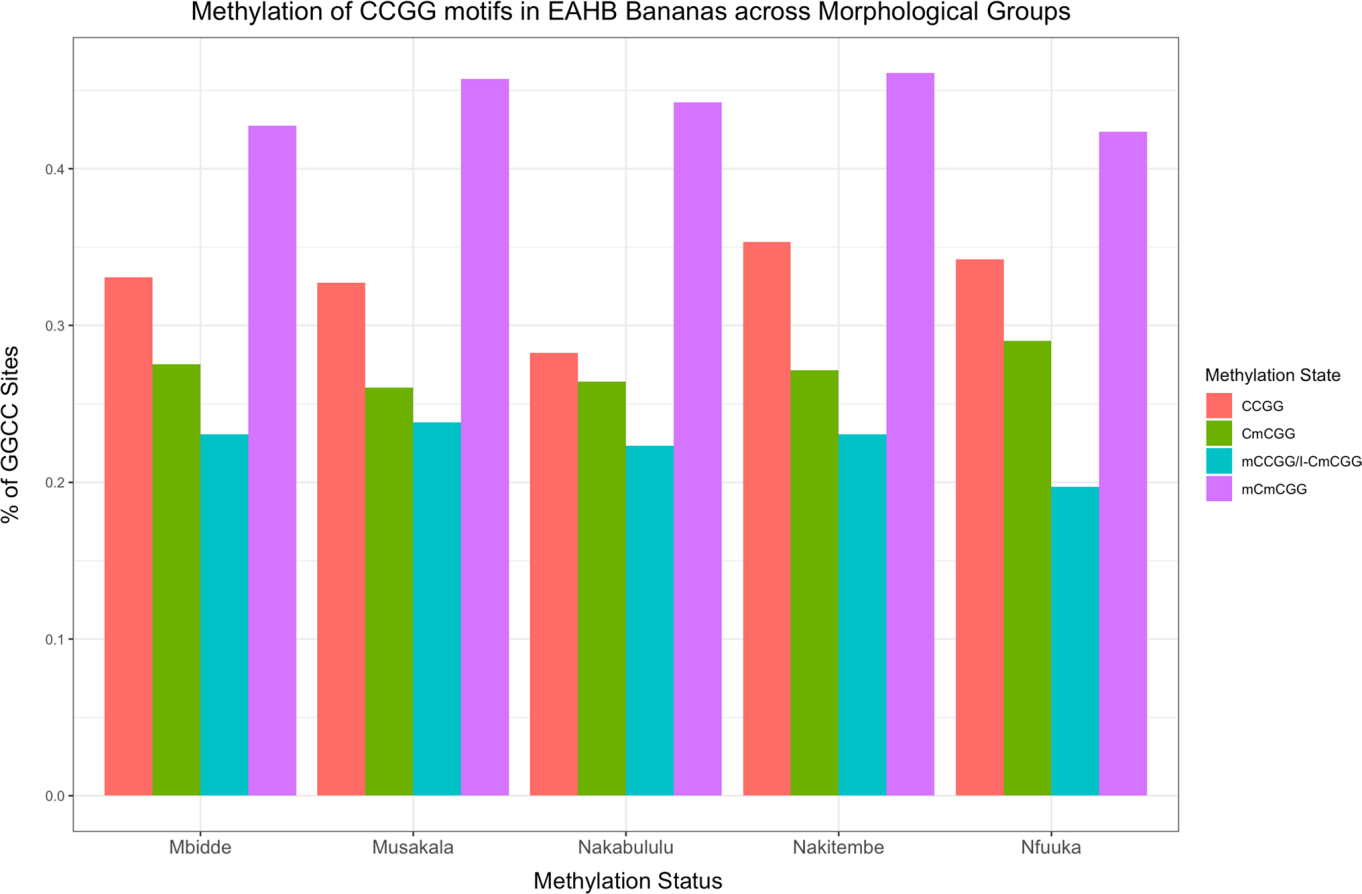
Proportion of genome-wide cytosine methylation in 724 MsAFLP loci of 90 EAHB cultivars. Differences in the number of the four types of fragments scored in the 90 cultivars were significant (Kruskal–Wallis test *P* < 0.0001, *** and Dunn’s Multiple comparison test)

Within the EAHB populations, a total of 724 bands were identified from MSAP and were used to perform principal coordinate analyses (PCoA) to explore epigenetic structure between morphological groups. Of these bands, 622 corresponded to potentially methylation-sensitive loci (i.e. MSL) and 102 bands to non-methylated loci (i.e. NML). The MSL sites were polymorphic (52% of the total MSL; Shannon's Diversity Index (*I*) = 0.3015; SD: 0.2289) and varieties were spread across the PCoA (Fig. 1a) compared to the non-methylated loci (63% polymorphic bands, Shannon's Diversity Index (*I*) = 0. 1547; SD: 0. 0784 (Fig. 1b), reflecting the status of variation in the two profiles. Differences in variation in the MSL and NML were significant (Wilcoxon rank sum test with continuity correction; *W* = 13,501; *P* < 0.0001). Across all MSL, instances of non-methylation occurred at 21.39% of loci, mCmCGG methylation at 46.3%, CmCGG methylation at 14% and indeterminate methylation at 18.31% (Fig. 2).

### MSAP DNA methylation levels and patterns do not epigenetically differentiate the five major morphological groups of EAHBs

**Fig. 3:**
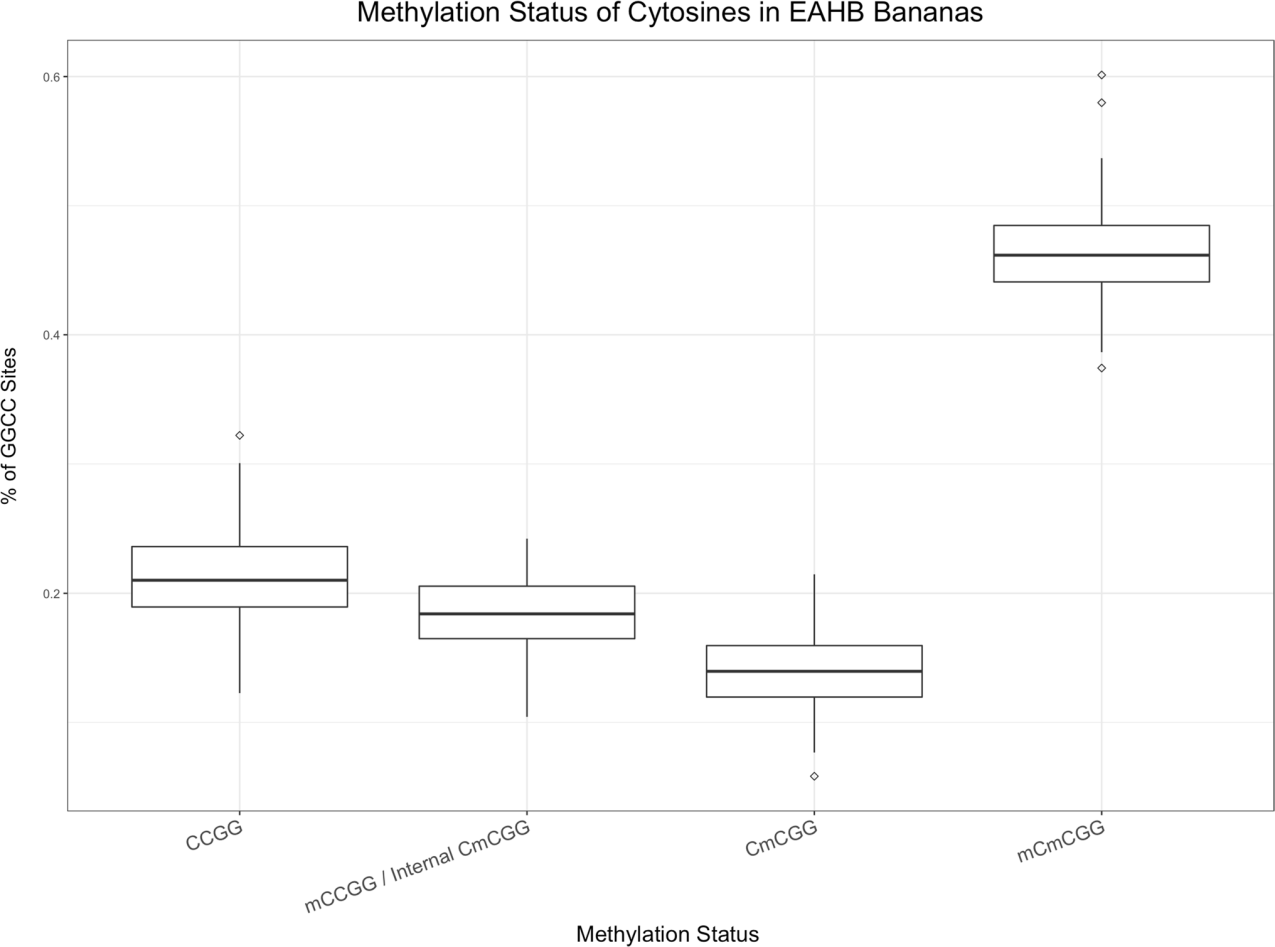
Relative proportions of CCGG methylation state (mCmCGG, CmCGG, CCGG and mCCGG/i-CmCGG) in 724 MSAP loci of cultivars in the five EAHB morphological groups

To determine whether epigenetic marks could distinguish between the five major recognized EAHB morphological groups, we compared their levels and patterns of DNA methylation (Fig. 3). The mean 5´-CCGG-methylation level among the morphological groups was 76.5% (of total loci scored) within a range of 76.2% (Musakala, the oldest cloneset) to 76.6% (Nakitembe). Statistical significance was observed in the proportions of each methylation state versus the other three states (Dunn`s Multiple comparison test, *P* < 0.0001 for all tests). The level of polymorphisms of the methylated loci did not differ significantly among the five morphological groups (Kruskal–Wallis *x*^2^ = 2.096, *P* = 0.7181; *x*^2^ = 6.349, *P* = 0.1745; *x*^2^ = 3.655, *P* = 0.4548 and *x*^2^ = 1.587, *P* = 0.8112 for CCGG sites, indeterminant internal sites, CmCGG sites and mCmCGG sites, respectively). AMOVA shows > 98% of the morphological groups’ genetic variation occurs within populations rather than between them. We conclude that patterns of total DNA methylation and DNA methylation polymorphisms show no meaningful epigenetic differences between morphologically distinct groups of EAHB cultivars.

### Geographic epigenetic differentiation of genetically identical EAHB populations

**Fig. 4:**
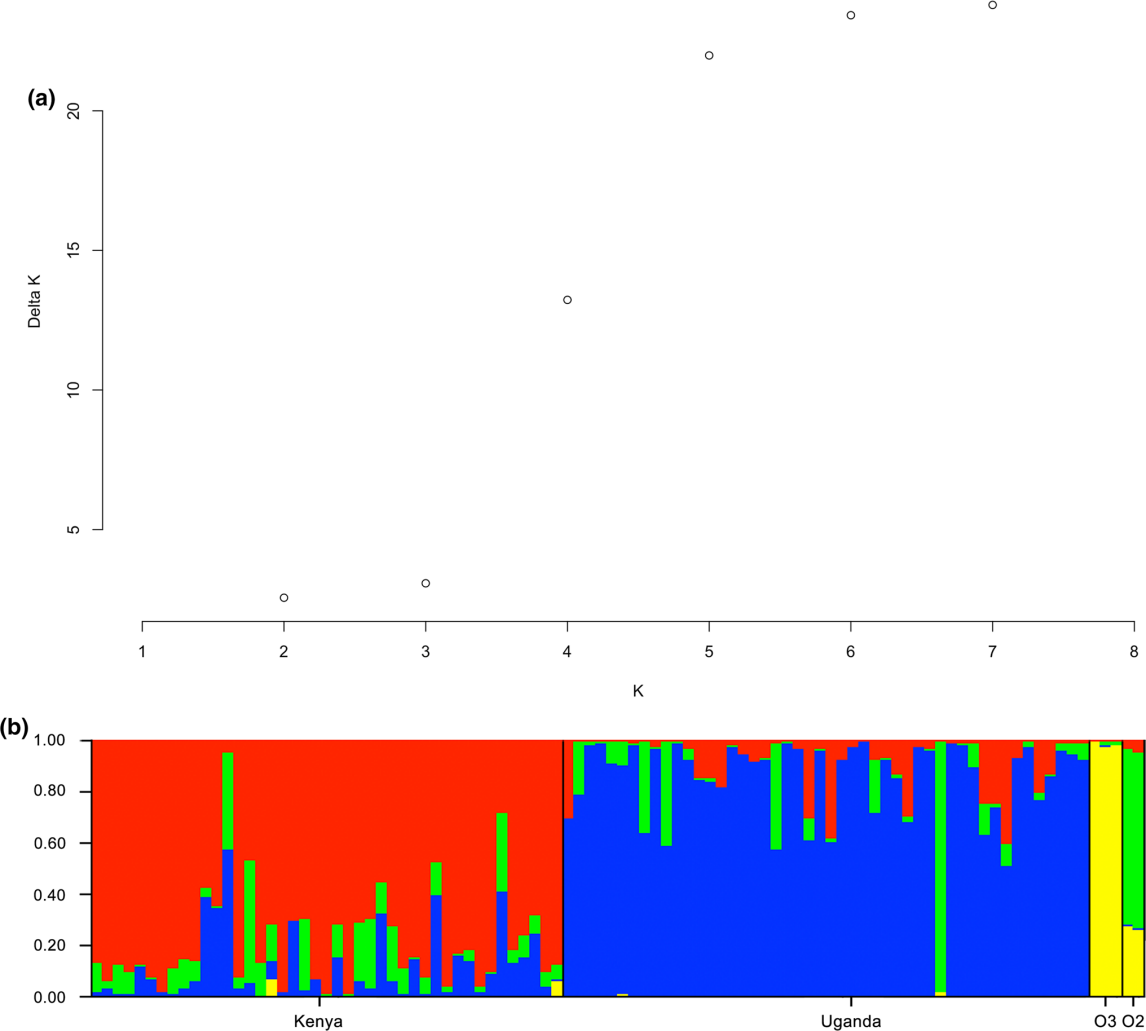
Epigenetic population structure and PCA of the EAHB. **a** Evanno test for determining the number of populations within the MSAFLP loci, **b** population structure was evaluated using STRUCTURE v2.3.3

As DNA methylation patterns only vary slightly between morphological groups, we considered what other factors might explain the epigenetic differentiation in EAHB. We considered the possibility of underlying epigenetic population structure not relating to morphology within the sampled bananas. Hence, we analysed the populations with Structure (Pritchard et al. 2010), assigning each cultivar to (epi)-populations (*K*), identified as migrants or admixed using MSAP multilocus genotype data independent of prior population information (Pritchard et al. 2000). The optimal number of Ks (1–10) was determined to be *K* = 5 (Fig. 4a) using both methylation-susceptible loci (MSL) and non-methylated loci (NML) datasets, as described in Methods. This resolved two groups indicative of the Dessert bananas and the Plantains, a group present in all groups except the Plantains (particularly in sample 61), and two groups that clustered according to whether the samples were taken from the Kenyan or Ugandan sites (Fig. 4b). We conclude that genetically near-identical EAHB cultivars are epigenetically distinguished on the basis of DNA methylation patterns that are associated with the two geographic regions (Uganda vs Kenya) from which they were sampled. To reflect this, outgroup 1, an EAHB from the Kenyan site of Unknown morphological grouping, was reclassified as part of the 90 cultivars, which henceforth are distinguished on the basis of sampling origin.

**Fig. 5:**
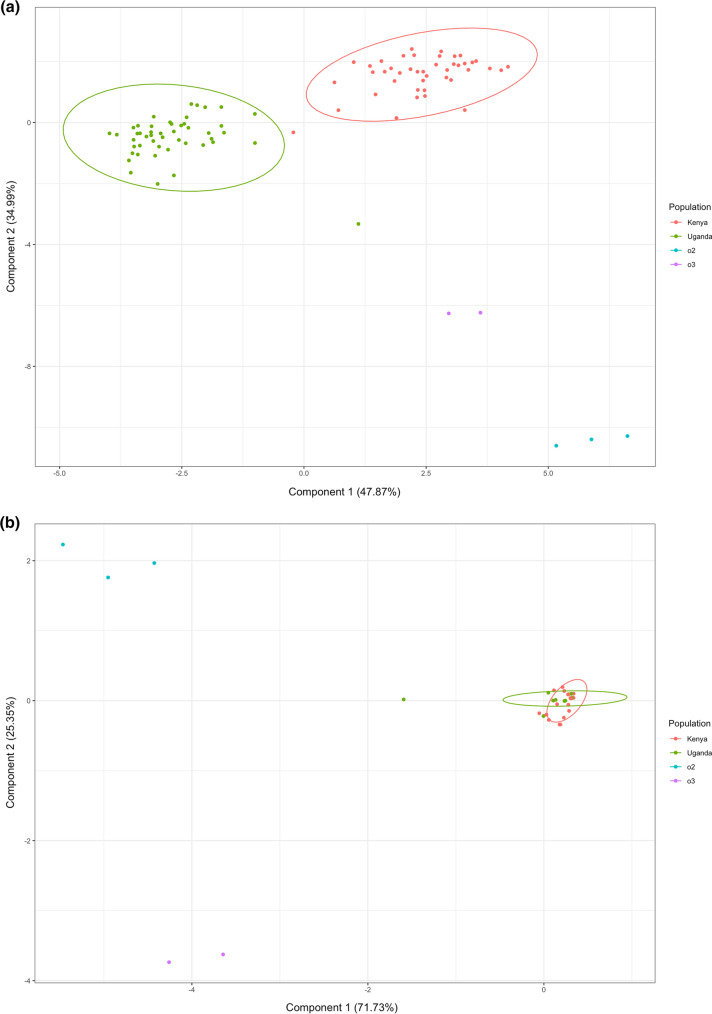
Between-group Eigen analysis (BPCA) based on the **a** MSL, **b** NML of the EAHB sampling sites (Kenya and Uganda) and outgroups (AAA—dessert banana, labelled as ‘O3’, and AAB—plantain, labelled as ‘O2’). Component 1 and Component 2 values show the contribution of the two principal components, summarizing the total variance of each data set

To further investigate the DNA methylation patterns in the five EAHB morphological groups, we generated a covariance matrix of their respective methylation profiles to perform between-group Eigen analysis (BPCA), with groups assigned based on the origin of the sample (Fig. 5). The first two axes of the BPCA for the MSL accounted for 82.9% of the total inertia between groups (Fig. 5a) while the first two axes of the BPCA for the NML summarized 97.2% of the inertia. In terms of the inertia of the PCoAs i.e. the variance both between and within groups; the MSL BPCA accounted for 21.6% of total inertia in the MSL, while the NML BPCA accounted for 49.2%. The MSL displayed separation of the two sampling sites along the first principal component and separation of the outgroups from the EAHBs along the second, while the NML only distinguished the outgroups from the EAHBs along its first two principal components (Fig. 5b). Comparisons of the methylated and non-methylated profiles between the sampling sites showed epigenetic differentiation (as measured by φst values), albeit with greater distance between the EAHB sites and the outgroups than within the EAHBs, while the NML showed very short distances between the EAHBs and considerable distances between the EAHBs and the outgroups (as well as between the two outgroups) (Table 1b). In contrast, the φst values for the morphological groups show little distance between themselves in either the MSL or NML, and very little significance in the comparisons measuring this distance. Distances between the morphological groups and the outgroups show significantly longer distances between the NML of the morphological groups and outgroups than the MSL. The unknown outgroup shows shorter and insignificant distance from the morphological groups, showing evidence it belongs among these groups. Dessert bananas and plantains are of roughly equivalent distance in the NML, but plantains show greater distance in the MSL profiles.

**Table 1:** Pairwise φ-ST values of MSL and NML comparing methylation-sensitive and methylation-insensitive differentiation among (a) the EAHB morphological groups and (b) the sampling site, with outgroups denoted as distinct from the sites

Cloneset A	Cloneset B	MSL	P (MSL)	NML	P (NML)
*a*					
Nakabululu	Nfuuka	0.002	0.343	0.004	0.352
Musakala	Nakitembe	0.001	0.390	− 0.019	0.902
Nakitembe	Nfuuka	0.023	0.061	0.016	0.196
Mbidde	Nfuuka	0.017	0.079	0.046	< 0.001
Musakala	Nfuuka	0.026	0.048	0.013	0.204
Mbidde	Nakabululu	0.022	0.076	0.008	0.241
Nakabululu	Nakitembe	0.017	0.120	0.003	0.370
Musakala	Nakabululu	0.023	0.091	0.001	0.430
Mbidde	Musakala	0.035	0.049	0.011	0.200
Mbidde	Nakitembe	0.034	0.033	0.017	0.149
O1 (Unknown)	O2 (Dessert)	0.662	0.327	0.539	0.338
O1 (Unknown)	O3 (Plantain)	0.611	0.262	0.594	0.247
O1 (Unknown)	Nfuuka	0.050	0.278	0.260	0.151
O1 (Unknown)	Nakitembe	0.089	0.196	− 0.077	0.199
O1 (Unknown)	Nakabululu	0.087	0.210	0.342	0.215
O1 (Unknown)	Mbidde	0.031	0.403	0.235	0.346
O1 (Unknown)	Musakala	0.122	0.371	0.184	0.251
O2 (Dessert)	O3 (Plantain)	0.656	0.102	0.626	0.102
O3 (Plantain)	Nfuuka	0.560	< 0.001	0.878	< 0.001
O3 (Plantain)	Nakitembe	0.522	0.002	0.761	0.001
O3 (Plantain)	Nakabululu	0.561	< 0.001	0.873	< 0.001
O3 (Plantain)	Mbidde	0.546	< 0.001	0.854	< 0.001
O3 (Plantain)	Musakala	0.611	0.001	0.841	0.002
O2 (Dessert)	Nfuuka	0.403	0.004	0.887	0.003
O2 (Dessert)	Nakitembe	0.353	0.008	0.765	0.009
O2 (Dessert)	Nakabululu	0.398	0.006	0.885	0.005
O2 (Dessert)	Mbidde	0.382	0.005	0.864	0.005
O2 (Dessert)	Musakala	0.376	0.008	0.852	0.008
*b*					
Kenya	O2 (Dessert)	0.555	< 0.001	0.856	< 0.001
O3 (Plantain)	Kenya	0.402	< 0.001	0.858	0.001
Kenya	Uganda	0.195	< 0.001	0.016	0.013
O2 (Dessert)	O3 (Plantain)	0.656	0.102	0.626	0.102
Uganda	O2 (Dessert)	0.595	< 0.001	0.866	< 0.001
O3 (Plantain)	Uganda	0.450	< 0.001	0.870	< 0.001

### Epigenetically divergent genetic clones and selection of East African Highland banana cultivars

Heritable epigenetic modifications can have morphological, physiological and ecological consequences, including across generations and geographies. Our previous studies have shown that EAHBs are genetically uniform, arising from a single ancestral clone that has since been vegetatively propagated and gave rise to the morphology-based EAHB germplasm collections that exist today (Kitavi et al. 2016). The morphological characteristics that differentiate EAHB cultivars must likely arise as a result of somaclonal mutations, mitotically inherited epigenetic mutations and/or genotype-by-environment (or epigenotype-by-environment) interactions. The epigenetic variation we have detected across the different EAHB cultivars (which are near isogenic) raises the possibility that heritable epigenetic mutations could be the source of phenotypic variation for new EAHB cultivars selected by farmers.

**Fig. 6:**
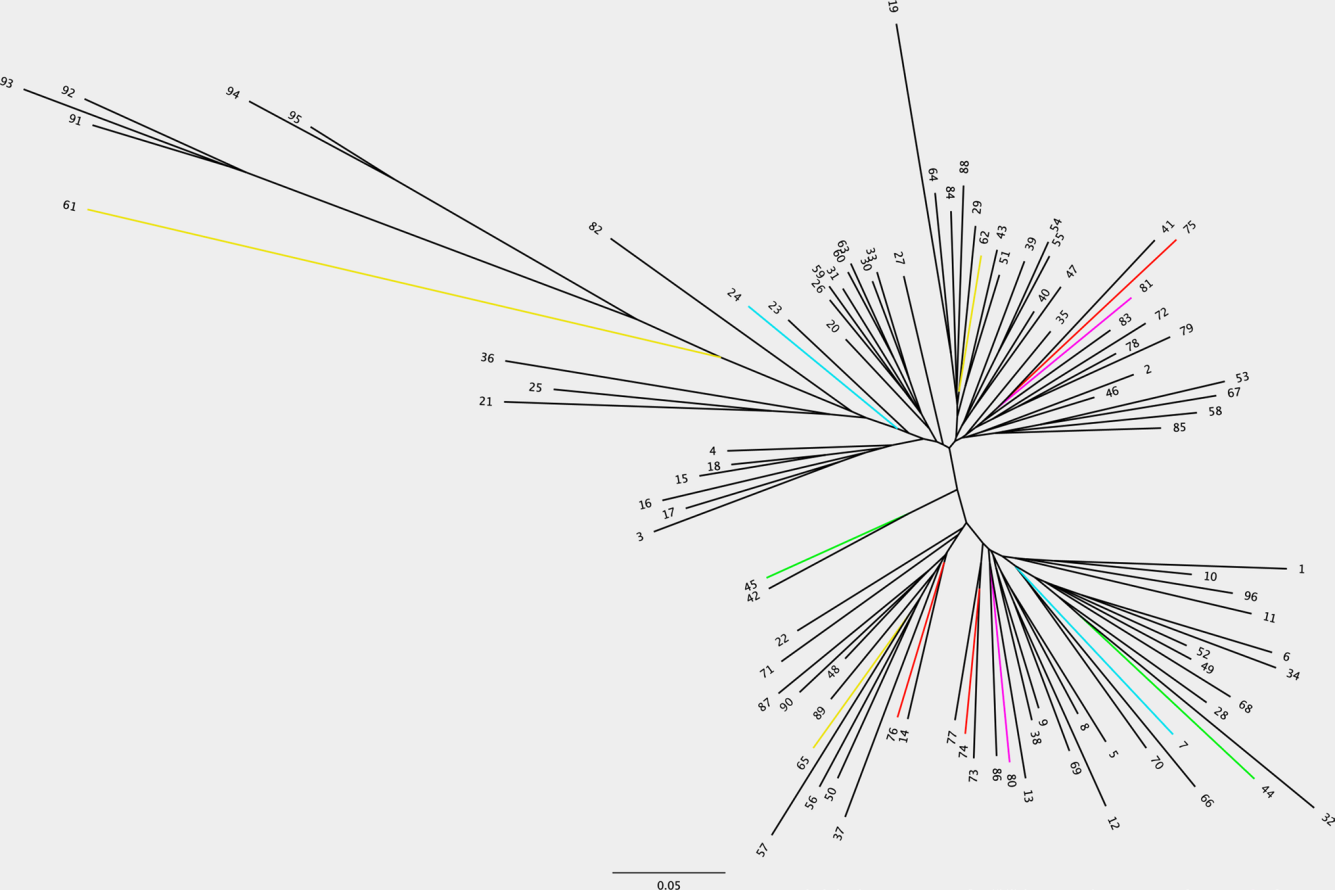
Neighbour joining (NJ) tree showing phylo-epigenetic differences between EAHB cultivar clones, EAHB clone variants and EAHB lineage clones raised in same field or in different fields; numbers in black at the tips of branches relate to cultivars listed in Supplementary Table 1

To investigate this possibility, we constructed a neighbour joining (NJ) tree representing phylo-epigenetic differences in clones grown in different fields, represented in different colours in Fig. 6 (clones 7 and 24 in blue, 45 and 44 in light green, 80 and 81 in pink; cultivar names in Supplementary Table 1), variants of the same clone growing in the same field (61, 62 and 65, all yellow) and lineage clones (mother plant 74, two sister clones, 75 and 76 all red). Clones 75 and 76 are derived from the same mother so should genetically be considered as clones, but differ in the phenotype of their rachis, prompting farmers to consider them as two different cultivars. Intriguingly, the NJ tree indicates the existence of epigenetic differentiation among groups of genetically identical EAHB clones with phenotypic differences. Consider, for example, the distance between 75 and 76: the former remains tightly clustered with 74, its mother clone, while 76 diverges. Similarly, 80 and 81 are even further apart despite being sister clones and therefore genetically identical. We conclude that environmental factors (including farmer selection) experienced by the clones during their cultivation generate differences in their DNA methylation, including in groups of clones that display agriculturally relevant phenotypic differences.

### Meiotic and mitotic transgenerational inheritance of cytosine methylation patterns in EAHB

**Fig. 7:**
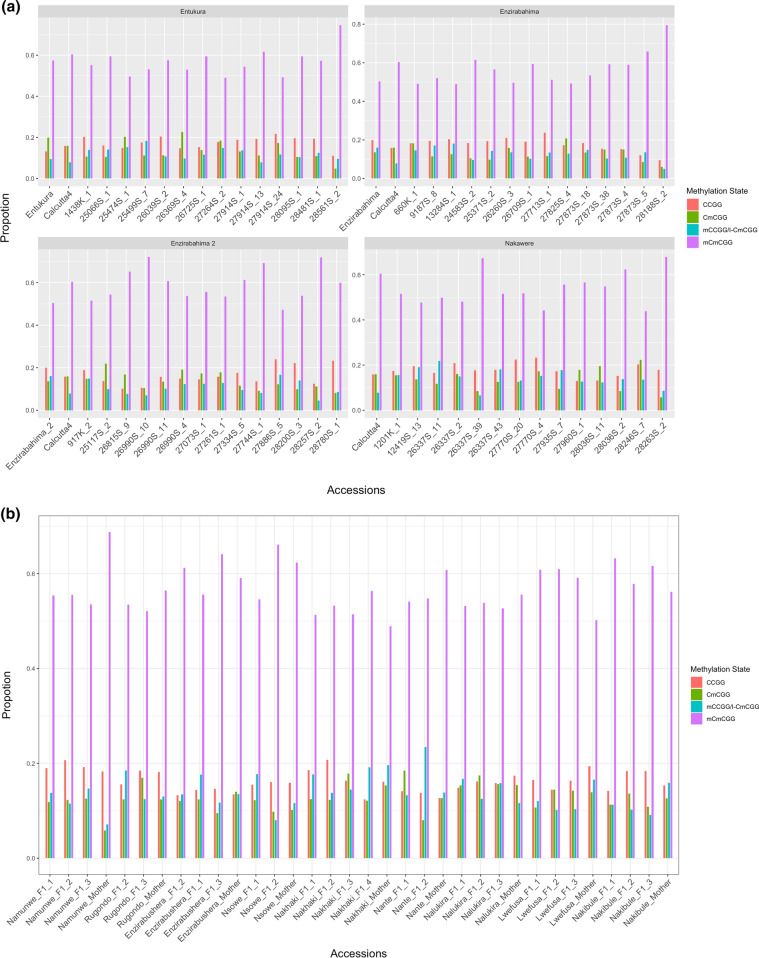
Mean percentage of CCGG methylation states sites at 1868 loci assessed across **a** four sexual families, comprising *F*_0_ (parents), *F*_1_s (tetraploids from a cross between wild calcutta4 and maternal landrace triploid EAHB) and *F*_2_s hybrids (triploids derived from a cross of a tetraploids with improved male diploids) and **b** asexual families represented by the mother plant and first cycle clonal plantlets derived through sucker vegetative propagation

DNA methylation can affect chromatin structure and transcriptional regulation and thereby impact on biochemical and/or morphological phenotypes. However, for DNA methylation-associated phenotypes to persist it requires that the responsible DNA methylation states (epialleles) are heritable, either meiotically (e.g. via seeds) or mitotically (e.g. via vegetative propagation). To determine whether the differences in DNA methylation observed between EAHB clones are heritable and thus able to contribute to adaptation of cultivars, we performed a further MSAP analysis to investigate DNA methylation levels and CCGG patterns in groups of parents and their meiotic (seed) and mitotic (vegetative propagated) progeny. Specifically, we generated sexual (meiotically derived) families by crossing four parental varieties of EAHB with a wild diploid cultivar, Calcutta4, as shown in Fig. 7a, to generate four sets of *F*_1_ offspring which were then backcrossed to generate divergent *F*_2_ offspring; 13 of these were randomly selected for analysis, giving 52 *F*_2_ in total. In addition, asexual (clonal) vegetatively propagated families were assessed from nine groups of clonally propagated parents and between two and four of their progeny. DNA methylation profiles were then analysed in all these meiotic and mitotic families using 10 primer combinations.

A total of 1868 loci were analysed of which 98% were methylated (MSL), with the remainder non-methylated (NML); the error rate per primer combination was 0.05. As before, the MSL was found to be significantly more diverse than the NML (Shannon's Diversity Index (*I*) = 0.3345; SD 0.2057 vs I = 0.2060; SD 0.076; Wilcoxon rank sum test with continuity correction W = 36,128; *P* < *P* = 0.00156). When progeny produced by the sexual (seed, meiotic) and asexual (clonal, mitotic) modes of propagation was compared, both were found to be highly polymorphic in both their MSL and NML fractions: 85% and 74% for MSL in the sexual and asexual progeny and 100% and 87% in the NML. In all cases, these were significantly diverse by Shannon's Diversity Index (Table 2). A mantel test for correlation among the MSL and NML profiles of the two reproductive methods shows no significant correlation in the meiotic populations while small amount of significant correlation is seen in vegetative propagated populations (Table 2; *r* = 0.13 and *P* = 0.092 vs *r* = 0.276; and *P* = 0.018), suggesting that meiotic recombination led to segregation of these loci.

**Table 2:** Methylation level and status in meiotic families versus the vegetative clones. Number and frequency of variant methylation patterns, Shannon's Diversity Index (*I*) and φ-ST of methylation susceptible loci (MSL) and nonmethylation susceptible loci (NML) in sexual and asexual vegetatively propagated EAHB groups across 1805 loci

Index	Sexual families	Asexual families
Number of samples/individuals	60	34
Number of groups/populations	6	9
Number of methylation-susceptible loci (MSL)	1837	1830
Number of non-methylated loci (NML)	31	38
Number of polymorphic MSL	1558 (85% of 1668)	1347 (74% of 1830)
Number of polymorphic NML	31 (100% of 31)	33 (87% 38)
Shannon's Diversity Index (*I*) MSL	0.3611 (SD; 0.1994)	0.3612 (SD; 0.1919)
Shannon's Diversity Index (*I*) NML	0.1888 (SD; 0.0737)	0.2110 (SD; 0.0672)
φ-ST MSL	0.03968 (*P* < 0.0001)	0.0921 (*P* < 0.0001)
φ-ST NML	0.06185 (*P* = 0.0266)	0.0502 (*P* = 0.051)
Mantel (*r*) correlation of MSL/NML	0.1302 (*P* = 0.0899)	0.3129 (*P* = 0.003)

The levels of CCGG-methylated sites in the parental lines (EAHB females; triploids and Calcutta 4; diploid), the *F*_1_ (tetraploids) and *F*_2_ (triploids) were then compared (Fig. 7a). Using Dunn’s test of multiple comparison, no significant variation in methylation levels as determined by MSAP was detected between any of the generations used to generate offspring meiotically within families derived from an *F*_0_ parent. In addition, no variation was found in the levels of each type of methylation across all families. A lack of significance is also observed with respect to differences in methylation levels of vegetatively propagated offspring and their parents, and this observation holds true both within and between vegetatively propagated families (Fig. 7b). Comparing the generations across the two subpopulations shows no significant difference in the proportions of loci occupying each of the methylation states between the sexual and asexual populations.

### Heritability of methylated loci in meiotic offspring versus mitotic offspring of East African Highland bananas

Methylated states (i.e. CCGG, CmCGG, mCmCGG and indeterminate methylated sites) present in the parents and their corresponding offspring were regarded as shared and therefore as potentially stably inherited. Methylation occurring in the offspring and not in their parents was regarded as novel. Loss of methylation was considered as methylation present at a locus in the parents that was missing in their respective offspring. Significant differences in abundances of the methylation states were not detected between parent(s) and subsequent generations using one-way ANOVA and Tukey’s test. Of note is an inability of this analysis to completely rule out novel epigenome reorganization that coincidentally matches the state of the parent. Efforts were made to account for de novo methylation states in the *F*_2_ that happened to match the *F*_1_ father, where analysis shows evidence for biased inheritance of one site type over others in certain contexts. However, none of the sites analysed offer convincing evidence of methylation-sensitive loci that are stably inherited in unique contexts.

**Fig. 8:**
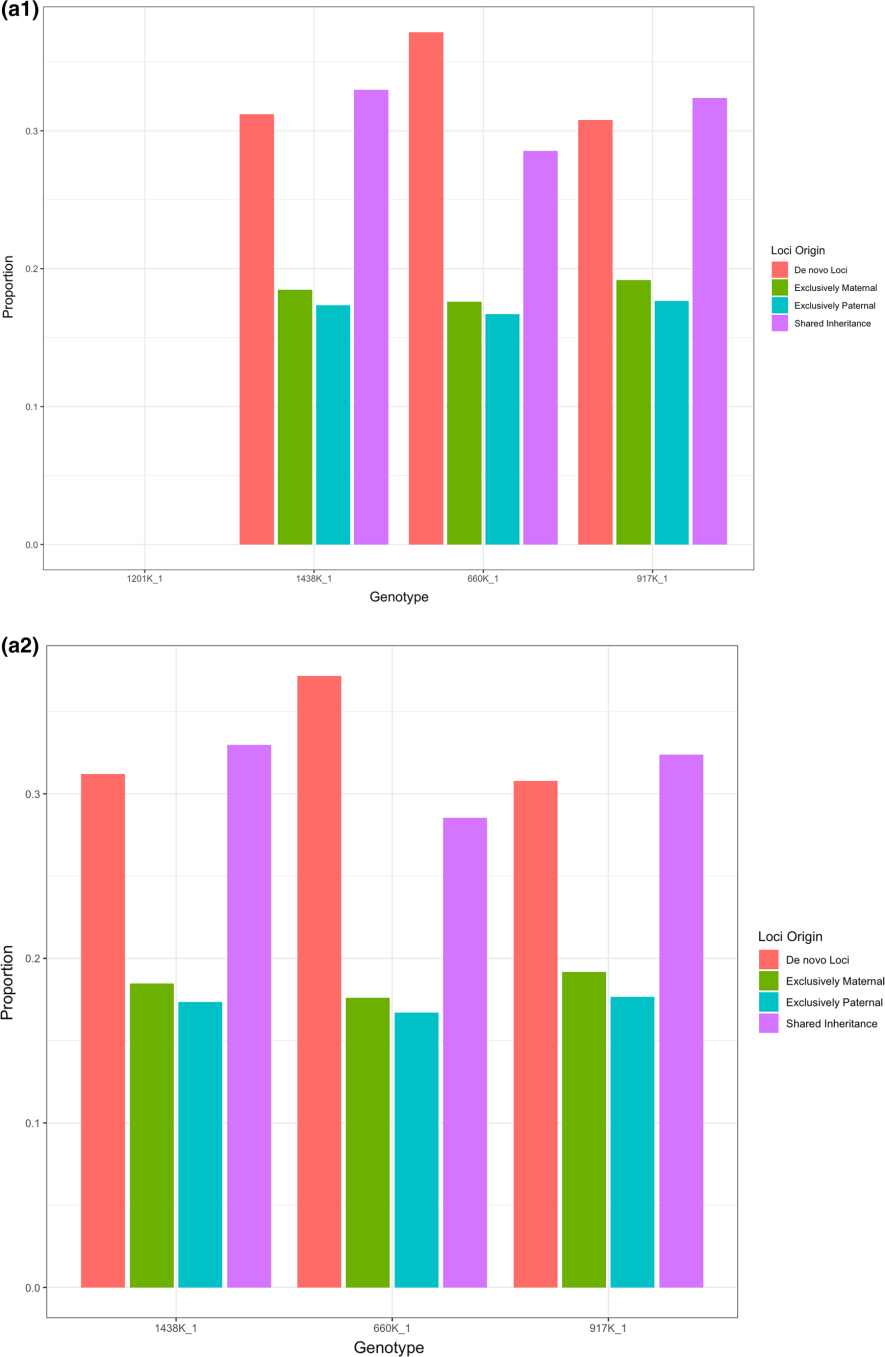

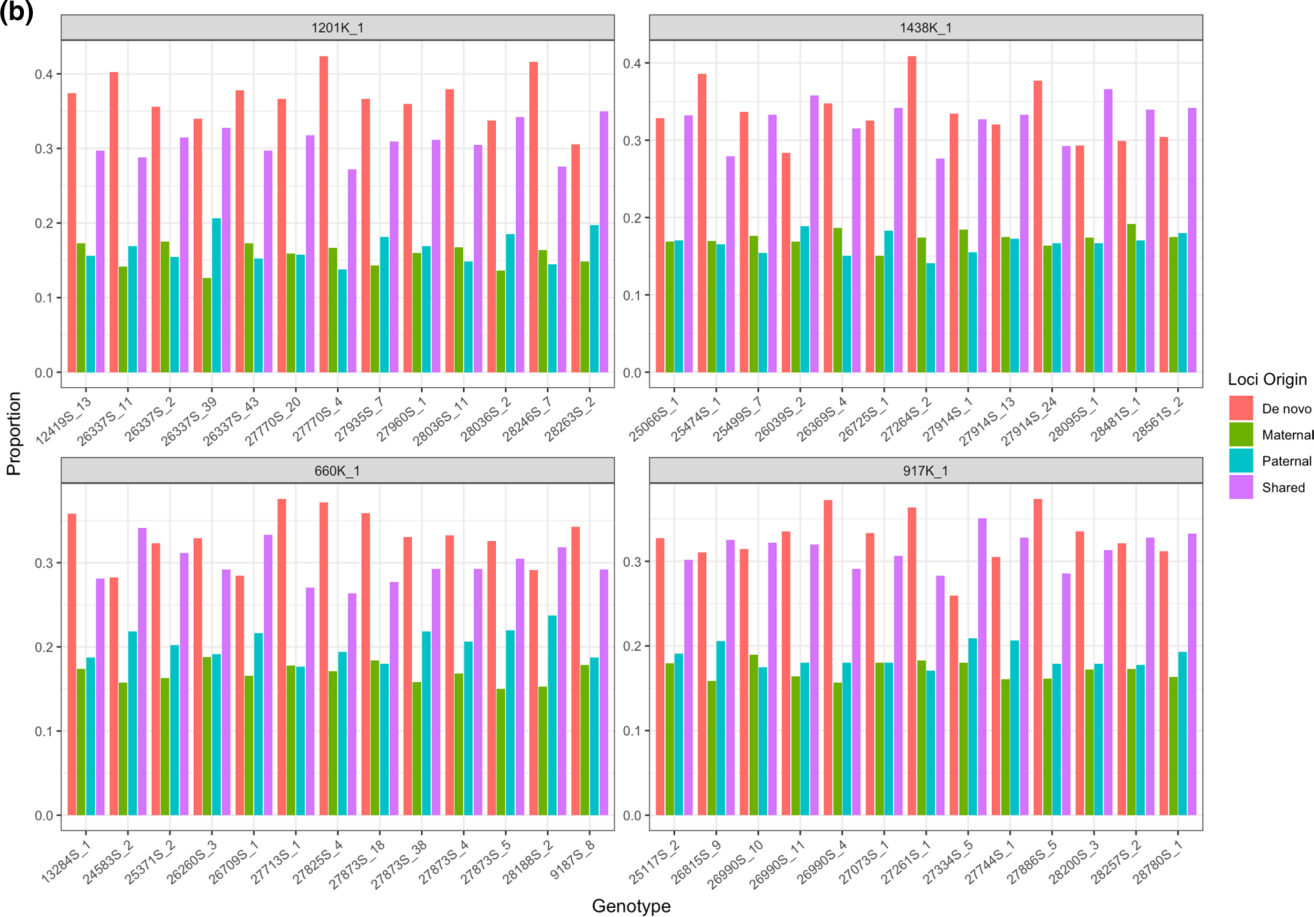
Total number of loci inherited by **a** the tetraploid *F*_1_s (1438-1, 660K-1, 917K-2 and 1201K-1) from the *F*_0_ generation and **b** the triploid *F*_2_s from the *F*_1_ generation in sexual families in terms of locus origin

**Fig. 9:**
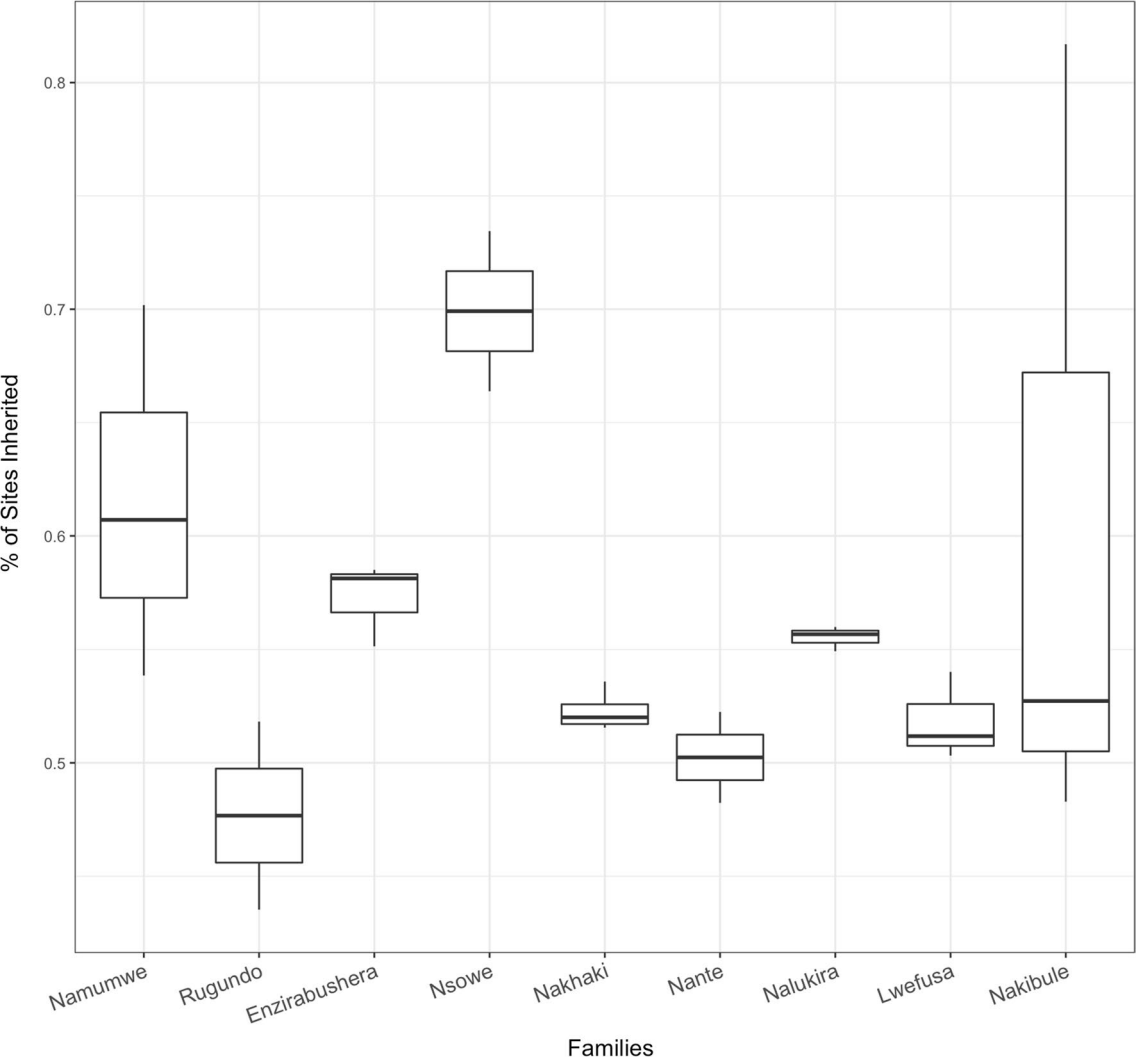
Comparison of inheritance of CCGG site methylation state between sucker/plantlet clones families derived from nine different mother plants (in the asexual (vegetative) families 7–15; Supplementary Fig.1). *Y*-axis represents the proportion of loci which match the mother plant

In the meiotic families, the probability of methylation patterns being shared between both parents and *F*_1_ offspring was 34.1%, with loci exclusive to the maternal parent comprising 16.7% and paternally exclusive loci being shared with 18% of loci (Fig. 8). Analysis pertaining to inheritance from the *F*_0_ generation is only available for three of the four families, as no data exist for the methylation profile of the Nakawere *F*_0_ parent. In contrast, for the vegetatively propagated asexual families, the proportion of methylated loci shared between parents and progeny ranged from 47.7 to 69.9% across the families, but with no significant difference detected between the vegetatively propagated families (Fig. 9) The overall rate for inheritance in mitotically reproducing families was 56.2%.

Analysis of the DNA methylation patterns of the parents and their offspring revealed patterns that were apparent across the set of loci as a whole but not evident at the individual level. Inherited loci in the *F*_1_ generation can be attributed to either a single parent, both parents or neither, while the *F*_2_ generation can be attributed to either having been inherited solely from the *F*_0_ maternal parent or the *F*_1_ parents, or shared inheritance from a combination of these individuals. No significant differences were detected in comparisons between the *F*_1_ progeny and the *F*_0_ parents (Table 3), but differences that become significant in the *F*_2_ generation are present. Examining inheritance in the *F*_2_ (Table 4) in terms of family revealed that the mean proportion of loci in the *F*_2_ generation shared with the *F*_0_ parents, as determined by presence in the *F*_0_ and *F*_1_, was significantly greater in the two Enzirabahima families than in the Entukura family. Consequently, the Entukura had a greater inheritance of novel loci from the *F*_1_ maternal genome then that of the other two families. Testing for significant difference between methylation states in terms of the attributable sources of inheritance showed that methylated loci are disproportionately inherited at loci where both parents are also methylated at the loci, while inheritance of non-methylated states seems to prevail at loci that either matched to only one parent or were novel within that individual. Analysis was also carried out at the level of individual loci to determine whether a family-specific pattern of inheritance could be observed across multiple generations. For each family, 9–15 loci in the *F*_2_ generation were found to be have a significantly greater inheritance of a locus in the *F*_1_ maternal genome in comparison to every other family. Each of these loci, however, is methylated and is not always inherited from the *F*_0_ generation. To summarize, a significant difference in the prevalence of mCmCGG loci over any other loci appearing in both parent and offspring can be detected in the meiotically generated families, but to what degree genuine inheritance as opposed to spurious matching of the most prevalent of potentially freely changing loci is occurring is unclear.

**Table 3:** Pairwise comparison of shared loci between the *F*_0_ and *F*_1_ in terms of family and methylation state: All values are *P* values from Dunn’s test of multiple comparison

Comparison	*F*_0_ M	*F*_0_ P	*F*_0_ M + P	*F*_1_
Entukura–Enzirabahima 1	1.0000 (+/−)	1.0000 (+/−)	0.6072 (+/−)	0.0558 (−/+)
Entukura–Enzirabahima 2	1.0000 (+/−)	1.0000 (+/−)	0.8655 (+/−)	0.1551 (−/+)
Enzirabahima 1–Enzirabahima 2	1.0000 (+/−)	1.0000 (−/+)	0.8655 (−/+)	0.5563 (+/−)
mCmCGG–CCGG	1.0000 (−/+)	1.0000 (+/−)	0.5641 (+/−)	0.8462 (−/+)
mCmCGG–CmCGG	1.0000 (−/+)	1.0000 (−/+)	0.1045 (+/−)	0.8712 (−/+)
mCmCGG–mCCGG/i-CmCGG	0.6775 (−/+)	1.0000 (−/+)	0.1177 (+/−)	1.0000 (−/+)
CCGG–CmCGG	1.0000	1.0000 (−/+)	1.0000 (−/+)	1.0000 (+/−)
CCGG–mCCGG/i-CmCGG	1.0000 (−/+)	0.9374 (−/+)	1.0000 (+/−)	1.0000 (+/−)
CmCGG–mCCGG/i-CmCGG	1.0000 (−/+)	1.0000 (−/+)	1.0000 (−/+)	1.0000 (−/+)

(+/−) indicates that a larger portion of the former group of the comparison is shared with the loci type in question than the latter, while (−/+) indicates the reverse. *F*_0_ M—loci that match exclusively to the *F*_0_ mother, *F*_0_ P—loci that match exclusively to the *F*_0_ father, *F*_0_ M + P—loci that match to both *F*_0_ mother and *F*_0_ father, *F*_1_—de novo loci in the *F*_1_ progeny

**Table 4:** Pairwise comparison of shared loci between the *F*_0_, *F*_1_ and *F*_2_ in terms of family and methylation state: All values are *P* values from Dunn’s test of multiple comparison

Comparison	*F*_0_ M	*F*_0_ M + P	*F*_1_ M	*F*_1_ P	*F*_1_ M + P	*F*_2_
Entukura—Enzirabahima 1	0.2080 (+/−)	**0.0132 (+/−)**	**> 0.0001 (−/+)**	0.4754 (−/+)	0.7861 (+/−)	1.0000 (−/+)
Entukura—Enzirabahima 2	0.0742 (+/−)	0.0917 (+/−)	**0.0001 (−/+)**	0.2598 (−/+)	0.7861 (−/+)	1.0000 (+/−)
Enzirabahima 1—Enzirabahima 2	0.5355 (+/−)	0.3949 (−/+)	0.0928 (+/−)	0.5941 (−/+)	0.5762 (−/+)	1.0000 (+/−)
mCmCGG–CCGG	**> 0.0001 (−/+)**	** > 0.0001 (+/−)**	0.1353 (+/−)	** > 0.0001 (−/+**)	** > 0.0001 (+/−)**	**0.0023 (−/+)**
mCmCGG–CmCGG	**0.0659 (−/+)**	** > 0.0001 (+/−)**	0.7432 (+/−)	** > 0.0001 (−/+)**	**0.0094 (+/−)**	** > 0.0001 (−/+)**
mCmCGG–mCCGG/i-CmCGG	0.0002 (−/+)	** > 0.0001 (+/−)**	**0.0403 (+/−)**	**0.0008 (−/+)**	**0.0283 (+/−)**	**> 0.0001 (−/+)**
CCGG–CmCGG	** > 0.0001 (+/−)**	**0.0160 (+/−)**	0.5635 (−/+)	0.7531 (+/−)	**0.0083 (−/+)**	0.0690 (−/+)
CCGG–mCCGG/i-CmCGG	**0.0005 (+/−)**	0.4431 (+/−)	0.7432 (+/−)	0.1904 (+/−)	**0.0017 (−/+)**	0.1762 (−/+)
CmCGG–mCCGG/i-CmCGG	0.0659 (−/+)	0.0868 (−/+)	0.2768 (+/−)	0.2465 (+/−)	0.6162 (−/+)	0.5703 (+/−)

Numbers highlighted in bold indicate significant differences

(+/−) indicates that a larger portion of the former group of the comparison is shared with the loci type in question than the latter, while (−/+) indicates the reverse. *F*_0_ M—loci that match to the *F*_0_ mother and are present in the *F*_1_ progeny, *F*_0_ M + P—loci that match to both *F*_0_ mother and *F*_0_ father which are present in the *F*_1_ progeny, *F*_1_ M—loci which were novel in the *F*_1_ mother that are shared with the *F*_2_ progeny, *F*_1_ P—loci which in the *F*_1_ father that are shared with the *F*_2_ progeny, *F*_1_ M + P—loci shared in the *F*_1_ mother and *F*_1_ father that are not shared with the *F*_0_ parents, *F*_2_—de novo loci in the *F*_2_ progeny

Investigation of the vegetatively propagated individuals in terms of methylation state showed a similar pattern of global methylation state occupation (Fig. 9), as is expected given the lack of significant difference between the mitotically reproducing and meiotically reproducing populations. For each family, no significance in inheritance of any methylation state over any of the others inherited was detected, but across all families a bias towards inheritance of mCmCGG loci could be discerned through multiple comparisons. Our results indicate that EAHBs can maintain many DNA methylation sites over generations when propagated either sexually or asexually, but maintain more DNA methylation sites when clonally (vegetatively) propagated. In addition, both modes of generation of offspring display losses and gains of methylated DNA sites, indicating that DNA methylation epiallelic diversity is dynamic during both sexual reproduction and asexual reproduction of EAHBs. Outside of the EAHB breeding programme, EAHBs have been vegetatively propagated by farmers for centuries, where the scale of such activities provides significant opportunity for epialleles affecting agronomic phenotypes of EAHBs to arise as geographically isolated, epigenetically distinct, varieties in different environments. Where such ‘functional’ epialleles are maintained over multiple vegetative generations, they could form a key underpinning component of the phenotypic diversity of EAHBs that is conserved in field genebanks.

## Discussion

The EAHB germplasm that is used as a basis for EAHB breeding and crop improvement efforts is stored in field germplasm collections in Africa and also in in vitro collections at the International Musa Germplasm Transit Centre (ITC), managed by Bioversity International and hosted at the Katholieke Universiteit Leuven (KU Leuven), Belgium. The main EAHB field genebanks are in Uganda (at the east and central African regional collection at Mbarara) and Kenya (at Kenya Agricultural Research Institute, now the Kisii Regional Research Center. We have previously demonstrated that the triploid East African Highland Banana (EAHB) genepool is genetically uniform arising from a single ancestral clone that underwent population expansion by vegetative propagation (Kitavi et al. 2016). Despite the genetic uniformity of the EAHB genepool, there is significant phenotypic diversity amongst the many farmer-selected landrace cultivars that dominate the Great Lakes region of East Africa. Such phenotypic diversity has prompted the establishment of germplasm collections and also the consideration of the Great Lakes region as a secondary centre of banana diversity (Tugume et al. 2002).

Based on 73 morphological traits, the EAHBs have been classified into groups (referred to as clone sets) to reflect their variation in vegetative structures, bunch, fruit and male bud. These are Nfuuka, Nakitembe, Musakala and Nakabululu (Karamura 1998). Sometimes cultivars of the cooking type become astringent and are no longer used for cooking but instead for making beer. These are referred to as Mbidde (Kitavi et al. 2016). As all farmer landraces of EAHBs have arisen from a single ancestral clone by vegetative (clonal) propagation, the extant phenotypic diversity of EAHBs may have arisen from rare somatic genetic mutations, mitotically heritable epigenetic modifications and/or genotype-by-environment interactions. In particular, the observation that EAHB types can transition over time from one morphotype to another (e.g. from a cooking banana to a beer banana) is suggestive of epigenetic changes underpinning such transitions, particularly where there is no geographic movement of the EAHB vegetative lineage.

In this follow-on study from Kitavi et al. (2016), we have used methylation-sensitive AFLP (MSAP) to analyse the DNA methylation patterns across 90 EAHB cultivars from both the Ugandan and Kenyan germplasm collections, and determined the fidelity of the inheritance of the DNA methylation marks across generations in clonal versus sexual lineages. The EAHB germplasm population analysed show high levels of genomic DNA methylation at 5´-CCGG-3´ sites. The proportion of CG methylation, which occurred at all mCmCGG and CmCGG sites, and a proportion of the indeterminate ones, was higher than the levels of CHG methylation. This pattern has also been observed in other plant genomes (Candaele et al. 2014; Lister et al. 2008; Osabe et al. 2014), although we note that CHG in EAHB still appears to be higher than in species such as *Arabidopsis thaliana* (Law and Jacobsen 2010). Notably, our phylogenetic approaches and structure analysis indicate that the Kenyan and Ugandan EAHB varieties cluster together, demonstrating that there are strong associations between DNA methylation patterns (epigenotypes) and the geographic areas they are sampled from.

The DNA methylation patterns of the EAHBs did not show any correlation to the morphological groups that are used to classify the EAHB germplasm collections. The lack of association could be due to the sample size (90 cultivars) or number of MSAP markers applied, or indeed indicate that there is no relationship between morphology-based classification and DNA methylation-based classification of the EAHBs. A more extensive epiGWAS analysis involving higher density epimarkers, greater numbers of EAHB clones, assessment of the morphological traits in terms of continuous variables instead of categorical morphotypes and large-scale reciprocal transplant studies would be needed to determine whether there is any significant association between epigenetic markers and the 73 traits that are used to classify EAHBs into the four morphotype clone sets. It is also important to note that DNA methylation is only one type of epigenetic mark, albeit a form of epigenetic mark that can be inherited mitotically or meiotically. While possible associations of the different clone groups with different histone modifications could be investigated, in the near term the DNA methylation polymorphism in EAHBs revealed in this study can provide an initial basis for epigenome based breeding for crops such as EAHBs which have limited genetic diversity, with further potential to develop epimarkers that are linked to traits of interest (Gallusci et al. 2017; Spillane and McKeown 2014; Springer 2013).

### Epigenetic differentiation of EAHB cultivars and morphotypes

The differentiation of methylation polymorphisms across the EAHB populations, together with the stability of methylation patterns over generations (vegetative or seed derived) within specific EAHB cultivars, suggests that epigenotypes of EAHBs may be subject to environmental and farmer selection effects. Any functional effects of epigenetic modifications over multiple generations will depend on what traits are affected by specific epigenetic modifications (Preite et al. 2018). Indeed, a stronger role for epigenetic variation contributing to morphological variation and environmental adaptation of plants is emerging (Furci et al. 2019; Kooke et al. 2015, 2019; Zhang et al. 2013, 2018), including for clonal plants, such as those which reproduce apomictically (de Carvalho et al. 2016; Sailer et al. 2016; Wilschut et al. 2016). Our study demonstrates that DNA methylation diversity is high in EAHB genotypes grown in the same environment over generations. Similar results have been reported in other cultivated plants, which could be suggestive of epigenetic variation compensating for lack of genetic variation (Osabe et al. 2014; Riddle and Richards 2005).

### Heritability and conservation of epigenetic diversity in East African Highland bananas

We observed a stable level of methylation states in the *F*_1_s (tetraploids) and *F*_2_ (triploids) in the sexual families compared with their parents, though loci differed greatly across progeny (Fig. 9). Our results indicate that sexually generated offspring of EAHBs generated through meiotic crosses in the breeding programme can transmit at least some of the DNA methylation patterns of the parental plants. In the instances where DNA methylation is linked to functional traits of interest to the EAHB breeding programme, these findings suggest that either functional epimutations or epimarkers associated with epigenetically derived traits could be integrated into the EAHB breeding programme.

Plant genetic resources conservation has transitioned over time from conservation of germplasm accessions that maximise diversity of morphological, to the incorporation of data on genetic diversity (i.e. from molecular markers) so that germplasm collections that maximize evolutionary history in a manageable number of accessions can be constructed. While the rationale for such approaches is sound, the emerging likelihood that epigenetic variants may exist in crop genepools that are of functional or agronomic importance provides a rationale for conservation of epigenotypes of importance to the crop’s biology, breeding efforts and the performance of cultivars in agricultural systems. Our results point towards location as a large contributor of the EAHB epigenome state, and as such ensuring epigenomes included in conservation efforts are sampled from a meaningful or diverse environment types could be important for maximizing epigenetic diversity conservation and sustainable use efforts.

In particular, we consider that the phenotypic diversity of clonal crops such as EAHBs or other allopolyploid crops, which have undergone severe genetic bottlenecks can have limited genetic diversity in their genepools, may be underpinned by heritable epimutations of agronomic importance. These may be meiotically or mitotically transmissible, depending on the propagation biology of the crop. Our results highlight that, in contrast to genetic diversity, there is significant DNA methylation diversity that has arisen between the EAHB varieties that have all arisen via vegetative propagation from a single ancestral clone. Moreover, our comparisons of asexual versus sexual reproduction of EAHBs indicate that DNA methylation heritability and dynamics between parent and offspring can be substantial, providing ample basis for the emergence of epigenotypes selected by environment and/or farmers (where epialleles are contributing to functionally important traits). While genetic similarity can be used as a basis to reduce numbers of accessions in germplasm collections, our results highlight that epigenome profiling of accessions can differentiate epigenotypes that should be retained in germplasm collections on the basis that they may harbour epialleles contributing to traits of importance to the agronomy and quality characteristics of the crop.

## Conclusions

Clonal crops that are vegetatively propagated have the potential for emergence of new variants due to genetic or epigenetic mutations. Some clonal crops (especially triploids or allopolyploids) have undergone severe genetic bottlenecks during their domestication and cultivation history. EAHBs present an excellent example of a clonal crop that has undergone such a genetic bottleneck, as the EAHB genepool is genetically uniform arising from vegetative propagation `lineages' from a single ancestral clone. Our results highlight that crops such as EAHBs can have genetically uniform yet morphologically diverse morphotypes (varieties) that display considerable epigenetic variation across epigenotypes and between parents and offspring. While our experiments do not demonstrate causality between epigenotypes and morphotypes, or between epialleles and traits, our results do highlight that epigenetic variation can provide a basis for conservation of morphologically distinct, yet morphologically diverse accessions in plant epigenetic conservation programmes. In addition, our results provide a foundational basis for the integration of DNA methylation profiling for germplasm conservation and harnessing epigenetics in breeding programmes for East African Highland bananas.

## Electronic supplementary material

Below is the link to the electronic supplementary material.

Supplementary file1 (DOCX 45 kb)

Supplementary Figure 1. Image of vegetative propagules taken from nine EAHB triploid mother plant cultivars (PDF 537 kb)

Supplementary Figure 2. Section of the cigar leaf of each East African Highland banana plant from which DNA was extracted for analysis (PDF 579 kb)

Supplementary Figure 3. Schematic of crossing schemes used to generate improved EAHB cultivars (PDF 131 kb)
