# Supplementary material for: Heritable epigenetic diversity for conservation and utilization of epigenetic germplasm resources of clonal East African Highland banana (EAHB) accessions
Source: Theor Appl Genet. 2020 Jul 27;133(9):2605–25. doi: 10.1007/s00122-020-03620-1 (PMC7419381; doi:10.1007/s00122-020-03620-1)
Supplement: Supplementary file 1 — Supplementary file1 (DOCX 45 kb) [file 122_2020_3620_MOESM1_ESM.docx]

**Supplementary files**

**Supplementary Table 1. Sample set 1 consists of Ninety East African Highland Banana cultivars and 6 outgroup varieties (3 plantain-AAB, 2 dessert- AAA bananas and one unknown genome) used to study polymorphism of DNA methylation patterns**

|  |  |  |  |  |
| --- | --- | --- | --- | --- |
| **Serial No.** | **Cultivar name** | **Morphological group** | **Country of collection** | **Accession ID** |
| 1 | CN111 | Mbidde | Kenya | KEN 158 |
| 2 | Inguba Ye embire | Mbidde | Uganda | MMC 325 |
| 3 | Endirira | Mbidde | Uganda | MMC 003 |
| 4 | Ensansa | Mbidde | Uganda | MMC 135 |
| 5 | Itarecia | Mbidde | Kenya | KEN 138 |
| 6 | KBU2 | Mbidde | Kenya | KEN 139 |
| 7 | Mpologoma | Mbidde | Kenya | KEN 155 |
| 8 | Msera | Mbidde | Kenya | KEN 161 |
| 9 | Mtagato | Mbidde | Kenya | KEN 135 |
| 10 | Mtagatu | Mbidde | Kenya | KEN 134 |
| 11 | Mtahato | Mbidde | Kenya | KEN 157 |
| 12 | Mtore | Mbidde | Kenya | KEN 157 |
| 13 | Mukoya | Mbidde | Kenya | KEN 162 |
| 14 | Mukubu | Mbidde | Kenya | KEN 142 |
| 15 | Nalukira | Mbidde | Uganda | MMC 012 |
| 16 | Nalwezinga | Mbidde | Uganda | MMC 073 |
| 17 | Namadhi | Mbidde | Uganda | MMC 012 |
| 18 | Nsowe | Mbidde | Uganda | MMC 321 |
| 19 | Oruhuna | Mbidde | Uganda | MMC 117 |
| 20 | Enyoya | Musakala | Uganda | MMC 052 |
| 21 | Kisansa | Musakala | Uganda | MMC 011 |
| 22 | Litambi | Musakala | Kenya | KEN 100 |
| 23 | Luwata | Musakala | Uganda | MMC 058 |
| 24 | Mpologoma | Musakala | Uganda | MMC 031 |
| 25 | Mrefu | Musakala | Kenya | KEN 081 |
| 26 | Mukazi Alanda | Musakala | Uganda | MMC 004 |
| 27 | Musakala | Musakala | Uganda | MMC 018 |
| 28 | Mutore | Musakala | Kenya | KEN 129 |
| 29 | Muvubo | Musakala | Uganda | MMC 010 |
| 30 | Namayovu | Musakala | Uganda | MMC 062 |
| 31 | Namunwe | Musakala | Uganda | MMC 032 |
| 32 | Ngombe | Musakala | Kenya | KEN 082 |
| 33 | Ngongo | Musakala | Uganda | MMC 524 |
| 34 | Turbo | Musakala | Kenya | KEN 113 |
| 35 | Kaitabunyonyi | Nakabululu | Uganda | MMC 006 |
| 36 | Butobe | Nakabululu | Uganda | MMC 029 |
| 37 | Jamaga | Nakabululu | Kenya | KEN 091 |
| 38 | Kaburut | Nakabululu | Kenya | KEN 114 |
| 39 | Kafunze | Nakabululu | Uganda | MMC 086 |
| 40 | Kazirakwe | Nakabululu | Uganda | MMC 072 |
| 41 | Kibuzi | Nakabululu | Uganda | MMC 020 |
| 42 | Mbululu NAK | Nakabululu | Kenya | KEN 083 |
| 43 | Mukubakonde | Nakabululu | Uganda | MMC 115 |
| 44 | Nakabululu | Nakabululu | Kenya | KEN 072 |
| 45 | Nakabululu | Nakabululu | Uganda | MMC 093 |
| 46 | Nakasabira | Nakabululu | Uganda | MMC 064 |
| 47 | Nakyetengu | Nakabululu | Uganda | MMC 008 |
| 48 | Nasirembe | Nakabululu | Kenya | KEN 071 |
| 49 | Ntobe | Nakabululu | Kenya | KEN 118 |
| 50 | Nzimola | Nakabululu | Kenya | KEN 116 |
| 51 | Salalugazi | Nakabululu | Uganda | MMC 088 |
| 52 | White Nakabululu | Nakabululu | Kenya | KEN 097 |
| 53 | Bikowekowe | Nakitembe | Uganda | MMC 198 |
| 54 | Engagara | Nakitembe | Uganda | TZA0059 |
| 55 | Entaragaza | Nakitembe | Uganda | TZA0100 |
| 56 | Kikuyu1 | Nakitembe | Kenya | KEN 076 |
| 57 | Kiganda Lusumba | Nakitembe | Kenya | KEN 074 |
| 58 | Luvuta | Nakitembe | Uganda | MMC 098 |
| 59 | Mbwazirume | Nakitembe | Uganda | MMC 021 |
| 60 | Nabuyobo | Nakitembe | Uganda | MMC 095 |
| 61 | Nakitembe Nakamari | Nakitembe | Uganda | MMC 332 |
| 62 | Nakitembe Nakawere | Nakitembe | Uganda | MMC 033 |
| 63 | Nakitembe Red | Nakitembe | Uganda | MMC 076 |
| 64 | Namaliga | Nakitembe | Uganda | MMC 007 |
| 65 | Namukhila | Nakitembe | Kenya | KEN 090 |
| 66 | Sialamule | Nakitembe | Kenya | KEN 084 |
| 67 | Bitambi | Nfuuka | Uganda | MMC 015 |
| 68 | Black Uganda green | Nfuuka | Kenya | KEN 028 |
| 69 | Bukamba | Nfuuka | Kenya | KEN 104 |
| 70 | Ekeganda | Nfuuka | Kenya | KEN 159 |
| 71 | Enjuta | Nfuuka | Kenya | KEN 124 |
| 72 | Enyeru | Nfuuka | Uganda | MMC 035 |
| 73 | Enzingo | Nfuuka | Kenya | KEN 120 |
| 74 | GNgiant | Nfuuka | Kenya | KEN 079 |
| 75 | Ingarama | Nfuuka | Uganda | MMC 279 |
| 76 | Ishighame | Nfuuka | Kenya | KEN 088 |
| 77 | Kiffuba | Nfuuka | Kenya | KEN 110 |
| 78 | Kiffuba | Nfuuka | Uganda | MMC 120 |
| 79 | Kulwoni | Nfuuka | Uganda | MMC 039 |
| 80 | Libukusu | Nfuuka | Kenya | KEN 107 |
| 81 | Lusumba | Nfuuka | Uganda | MMC 036 |
| 82 | Mtama | Nfuuka | Kenya | KEN 126 |
| 83 | Namwezi | Nfuuka | Uganda | MMC 022 |
| 84 | Ndibwabalangira | Nfuuka | Uganda | MMC 019 |
| 85 | Nfuuka | Nfuuka | Uganda | MMC 017 |
| 86 | Enzingo with Normal rachis | Nfuuka | Kenya | KEN 120 |
| 87 | Nyarluoratong | Nfuuka | Kenya | KEN 004 |
| 88 | Rwambarara | Nfuuka | Uganda | MMC 130 |
| 89 | Enzigo with Spiral rachis | Nfuuka | Kenya | KEN 120 var. |
| 90 | White Uganda green | Nfuuka | Kenya | KEN 028 var. |
| 91 | MunjuP | Unknown | Kenya | KEN 140 |
| 92 | Spambia4 | Plantain | Kenya | KEN 065 var. |
| 93 | Spambia5 | Plantain | Kenya | KEN 065 var. |
| 94 | Spambia7 | Plantain | Kenya | KEN 065 var. |
| 95 | Redgreen | AAA desert | Kenya | MMC 384 |
| 96 | Somatic green | AAA desert | Kenya | KEN 013 |

**Supplementary Table 2. Sample set 2 used for DNA methylation heritability study.** Sexual families are represented by S/No 1-60 and vegetative clones are represented by S/No 61-69. The number of 1^st^ cycle offspring in the vegetative clone families is represented by a superscript on each cultivar name**.**

| **S/No** | **Genotype**  **name** | **Description** | **Ploidy** | **Fparent** | **Mparent** | **Bunch character** | **Other analysis info** |
| --- | --- | --- | --- | --- | --- | --- | --- |
| 1 | 27770S-20 | F_2_ | 3x | 1201K-1 | C.V rose | inferior bunch | Pop1 |
| 2 | 27770S-4 | F_2_ | 3x | 1201K-1 | C.V rose | good bunch size | Pop1 |
| 3 | 27935S-1 | F_2_ | 3x | 1201K-1 | C.V rose | good bunch size | Pop1 |
| 4 | 28036S-11 | F_2_ | 3x | 1201K-1 | C.V rose | inferior bunch | Pop1 |
| 5 | 28036S-2 | F_2_ | 3x | 1201K-1 | C.V rose | good bunch size | Pop1 |
| 6 | 28246S-7 | F_2_ | 3x | 1201K-1 | C.V rose | good bunch size | Pop1 |
| 7 | 27935S-7 | F_2_ | 3x | 1201K-1 | C.V rose | inferior bunch | Pop1 |
| 8 | 26337S-11 | F_2_ | 3x | 1201K-1 | SH-3217 | good bunch size | Pop1 |
| 9 | 12419S-13 | F_2_ | 3x | 1201K-1 | SH-3217 | good bunch size | Pop1 |
| 10 | 26337S-2 | F_2_ | 3x | 1201K-1 | SH-3217 | good bunch size | Pop1 |
| 11 | 26337S-39 | F_2_ | 3x | 1201K-1 | SH-3217 | inferior bunch | Pop1 |
| 12 | 26337S-43 | F_2_ | 3x | 1201K-1 | SH-3217 | good bunch size | Pop1 |
| 13 | 28263S-2 | F_2_ | 3x | 1201k-1 | SH-3217 | good bunch size | Pop1 |
| 14 | 27914S-1 | F_2_ | 3x | 1438K-1 | C.V rose | good bunch size | Pop2 |
| 15 | 27914S-13 | F_2_ | 3x | 1438K-1 | C.V rose | good bunch size | Pop2 |
| 16 | 28095S-1 | F_2_ | 3x | 1438K-1 | C.V rose | inferior bunch | Pop2 |
| 17 | 27264S-2 | F_2_ | 3x | 1438K-1 | C.V rose | inferior bunch | Pop2 |
| 18 | 27914S-24 | F_2_ | 3x | 1438K-1 | C.V rose | good bunch size | Pop2 |
| 19 | 25066S-1 | F_2_ | 3x | 1438K-1 | Kokopo | inferior bunch | Pop2 |
| 20 | 25474S-1 | F_2_ | 3x | 1438K-1 | Kokopo | good bunch size | Pop2 |
| 21 | 26369S-4 | F_2_ | 3x | 1438K-1 | Long tavoy | inferior bunch | Pop2 |
| 22 | 28481S-1 | F_2_ | 3x | 1438K-1 | Malaccensis | inferior bunch | Pop2 |
| 23 | 28561S-2 | F_2_ | 3x | 1438K-1 | Malaccensis | inferior bunch | Pop2 |
| 24 | 26725S-1 | F_2_ | 3x | 1438K-1 | SH-3362 | good bunch size | Pop2 |
| 25 | 25499S-7 | F_2_ | 3x | 1438K-1 | SH-3142 | good bunch size | Pop2 |
| 26 | 26039S-2 | F_2_ | 3x | 1438K-1 | SH-3217 | inferior bunch | Pop2 |
| 27 | 24583S-2 | F_2_ | 3x | 660K-1 | 5610S-1 | inferior bunch | Pop3 |
| 28 | 26260S-3 | F_2_ | 3x | 660K-1 | 5610S-1 | good bunch size | Pop3 |
| 29 | 13284S-1 | F_2_ | 3x | 660K-1 | 9128-3 | good bunch size | Pop3 |
| 30 | 25371S-2 | F_2_ | 3x | 660K-1 | 9128-3 | good bunch size | Pop3 |
| 31 | 9187S-8 | F_2_ | 3x | 660K-1 | 9128-3 | good bunch size | Pop3 |
| 32 | 26709S-1 | F_2_ | 3x | 660K-1 | Calcutta 4 | inferior bunch | Pop3 |
| 33 | 27713S-1 | F_2_ | 3x | 660K-1 | Malaccensis | good bunch size | Pop3 |
| 34 | 27825S-4 | F_2_ | 3x | 660K-1 | Malaccensis | good bunch size | Pop3 |
| 35 | 27873S-18 | F_2_ | 3x | 660K-1 | Malaccensis | inferior bunch | Pop3 |
| 36 | 27873S-38 | F_2_ | 3x | 660K-1 | Malaccensis | good bunch size | Pop3 |
| 37 | 27873S-4 | F_2_ | 3x | 660K-1 | Malaccensis | inferior bunch | Pop3 |
| 38 | 27873S-5 | F_2_ | 3x | 660K-1 | Malaccensis | inferior bunch | Pop3 |
| 39 | 28188S-2 | F_2_ | 3x | 660K-1 | Malaccensis | inferior bunch | Pop3 |
| 40 | 25117S-2 | F_2_ | 3x | 917K-2 | 5610S-1 | good bunch size | Pop4 |
| 41 | 26815S-9 | F_2_ | 3x | 917K-2 | 5610S-1 | inferior bunch | Pop4 |
| 42 | 26990S-10 | F_2_ | 3x | 917K-2 | 5610S-1 | inferior bunch | Pop4 |
| 43 | 26990S-11 | F_2_ | 3x | 917K-2 | 5610S-1 | good bunch size | Pop4 |
| 44 | 26990S-4 | F_2_ | 3x | 917K-2 | 5610S-1 | inferior bunch | Pop4 |
| 45 | 27073S-1 | F_2_ | 3x | 917K-2 | 5610S-1 | good bunch size | Pop4 |
| 46 | 27744S-1 | F_2_ | 3x | 917K-2 | 5610S-1 | good bunch size | Pop4 |
| 47 | 27261S-1 | F_2_ | 3x | 917K-2 | Malaccensis | inferior bunch | Pop4 |
| 48 | 27334S-5 | F_2_ | 3x | 917K-2 | Malaccensis | inferior bunch | Pop4 |
| 49 | 27886S-5 | F_2_ | 3x | 917K-2 | Malaccensis | good bunch size | Pop4 |
| 50 | 28033S-3 | F_2_ | 3x | 917K-2 | Malaccensis | good bunch size | Pop4 |
| 51 | 28257S-2 | F_2_ | 3x | 917K-2 | Malaccensis | good bunch size | Pop4 |
| 52 | 28780S-1 | F_2_ | 3x | 917K-2 | Malaccensis | inferior bunch | Pop4 |
| 53 | 1438K-1 | F_1_ | 4x | Entukura | Calcutta4 | good bunch size | Pop5 |
| 54 | 660K-1 | F_1_ | 4x | Enzirabahima | Calcutta4 | good bunch size | Pop5 |
| 55 | 917K-2 | F_1_ | 4x | Enzirabahima | Calcutta4 | good bunch size | Pop5 |
| 56 | 1201K-1 | F_1_ | 4x | Nakawere | Calcutta4 | good bunch size | Pop5 |
| 57 | Calcutta4 | Parent (Male) | 2x |  |  | inferior bunch | Pop6 |
| 58 | Entukura | Parent (Female | 3x |  |  | good bunch size | Pop6 |
| 59 | Enzirabahima | Parent (Female) | 3x |  |  | good bunch size | Pop6 |
| 60 | Nakawere | Parent (Female) | 3x |  |  | good bunch size | Pop6 |

Note; Enzirabahima clones were used twice as a female parent; The superscript on clonal families represent the number of 1^st^ cycle offspring that could be found for each mother plant.

**Supplementary Table 3**: Sequences of adaptors, preselective and selective primers used. (F) = Forward primer; (R) = Reverse primer.

| **Category** |  | **Sequence (5’ to 3’)** |
| --- | --- | --- |
| **Adaptor sequences** | *Hpa*II/*Msp*I | (F) GACGATGAGTCTAGAA  (R) CTACTCAGATCTTGC |
|  | *Eco*RI | (F) CTCGTAGACTGC GTAC  (R) AATTGGTACGCAGTCTAC |
| **Pre-selective primer sequences** | *Eco*RI (E00) | GTAGACTGCGTACCAATTCA |
|  | *Hpa*II/*Msp*I (M00/H00) | ATCATGAGTCCTGCTCGGT |
| **Selective primers** |  |  |
| Combination 1 | E-ACA (NED) | GACTGCGTACCAATTCACA |
|  | HPA2-AGC | ATCATGAGTCCTGCTCGGAGC |
| Combination 2 | E-AGT (FAM) | GACTGCGTACCAATTCAGT |
|  | HPA2-ATC | ATCATGAGTCCTGCTCGGATC |
| Combination 3 | E-AGG (FAM) | GACTGCGTACCAATTCAGG |
|  | HPA2-AGT | ATCATGAGTCCTGCTCGGAGT |
| Combination 4 | E-AGC (NED) | GACTGCGTACCAATTCAGC |
|  | HPA2-ATT | ATCATGAGTCCTGCTCGGATT |
| Combination 5 | E-ACG (FAM) | GACTGCGTACCAATTCACG |
|  | HPA2-ACA | ATCATGAGTCCTGCTCGGACA |
| Combination 6 | E-ACC (NED) | GACTGCGTACCAATTCACC |
|  | HPA2-ACT | ATCATGAGTCCTGCTCGGACT |
