# Supplementary material for: Heritable epigenetic diversity for conservation and utilization of epigenetic germplasm resources of clonal East African Highland banana (EAHB) accessions
Source: Theor Appl Genet. 2020 Jul 27;133(9):2605–25. doi: 10.1007/s00122-020-03620-1 (PMC7419381; doi:10.1007/s00122-020-03620-1)
Supplement: Supplementary file 2 — Supplementary Figure 1. Image of vegetative propagules taken from nine EAHB triploid mother plant cultivars (PDF 537 kb) [file 122_2020_3620_MOESM2_ESM.pdf]

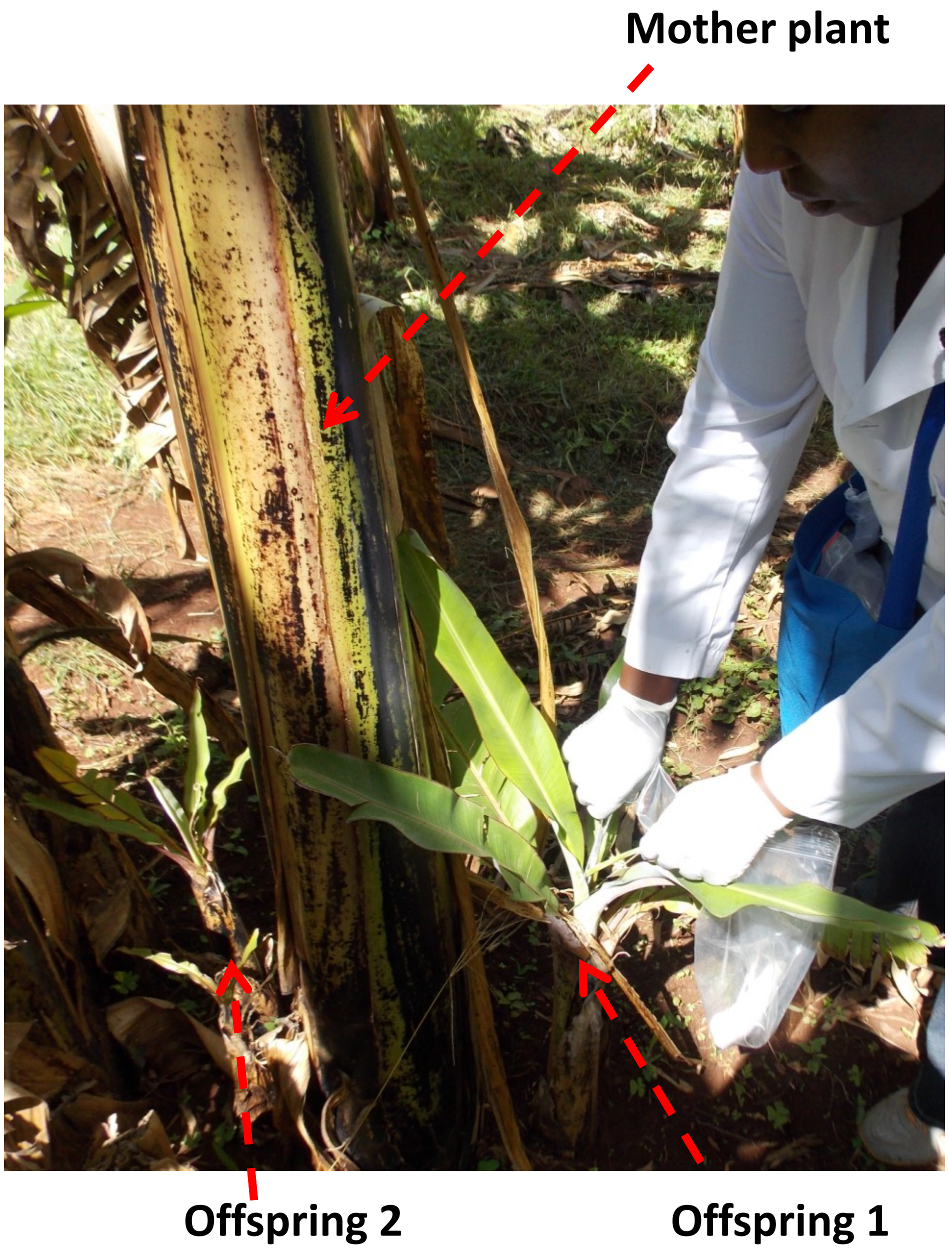

#	EAHB cultivar	Ploidy	No. of offsprings
1	Namunwe	3x	3
2	Rugondo	3x	2
3	Enzirabushera	3x	3
4	Nsowe	3x	2
5	Nakhaki	3x	4
6	Nante	3x	2
7	Nalukira	3x	3
8	Lwefusa	3x	3
9	Nakibule	3x	3
