# Supplementary figures and images for: Heritable epigenetic diversity for conservation and utilization of epigenetic germplasm resources of clonal East African Highland banana (EAHB) accessions
Source: Theor Appl Genet. 2020 Jul 27;133(9):2605–25. doi: 10.1007/s00122-020-03620-1 (PMC7419381; doi:10.1007/s00122-020-03620-1)

Section of a cigar  
leaf of the banana  
plant

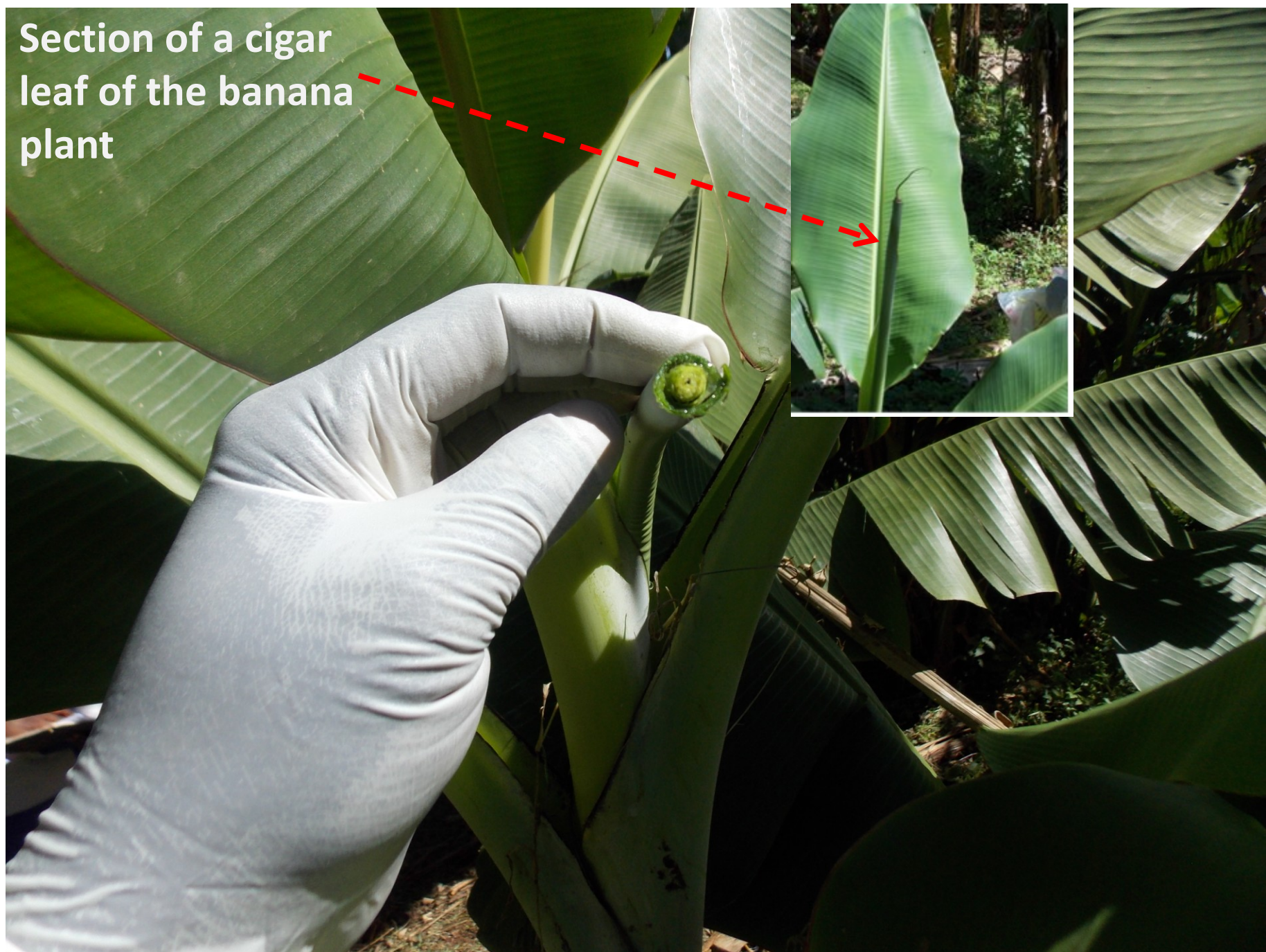

Supplement: Supplementary file 3 — Supplementary Figure 2. Section of the cigar leaf of each East African Highland banana plant from which DNA was extracted for analysis (PDF 579 kb) [file 122_2020_3620_MOESM3_ESM.pdf]

## Scheme on how crossing is done to generate improved EAHB cultivars

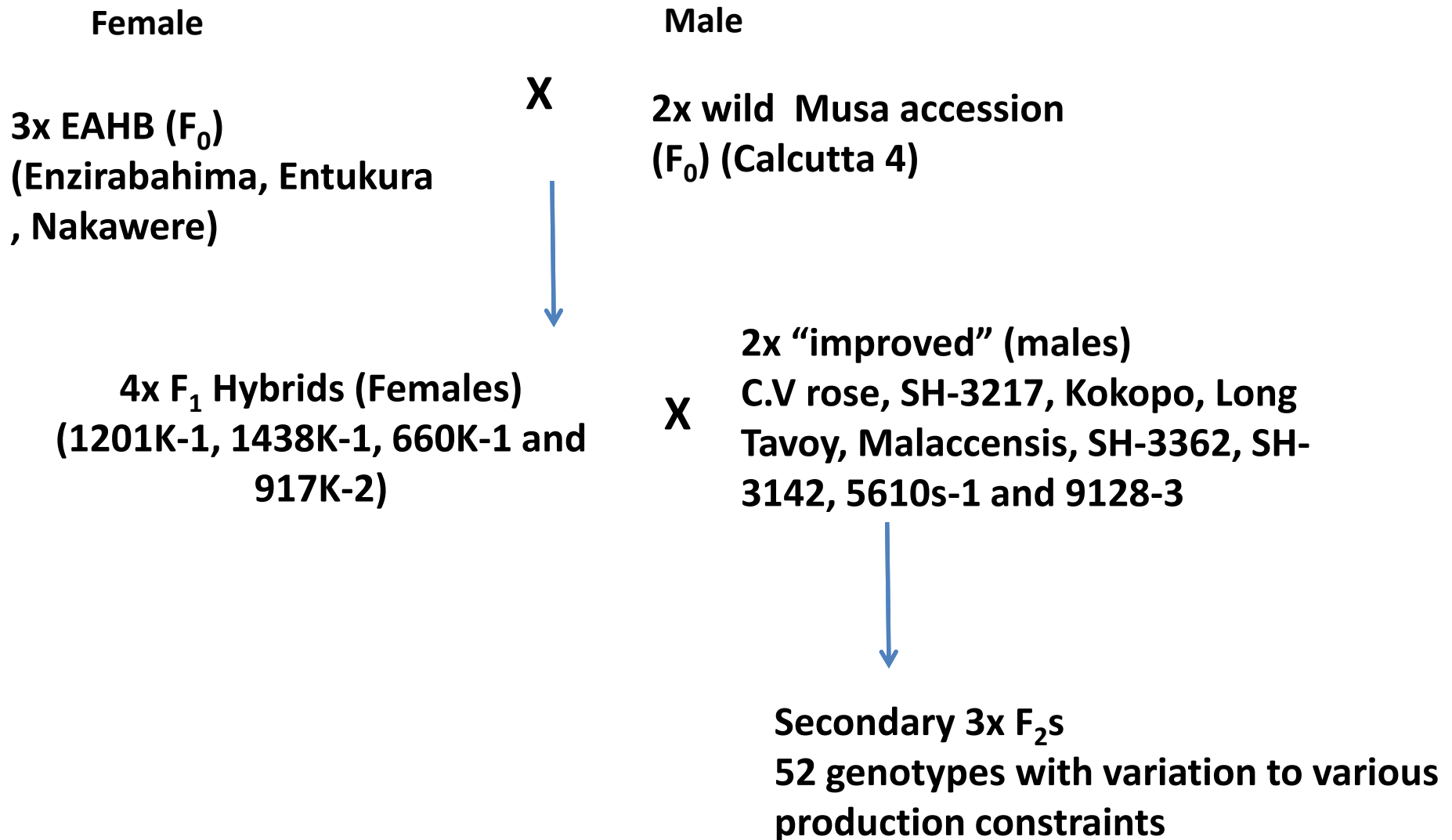

Supplement: Supplementary file 4 — Supplementary Figure 3. Schematic of crossing schemes used to generate improved EAHB cultivars (PDF 131 kb) [file 122_2020_3620_MOESM4_ESM.pdf]
